# A Second Mortuary Hiatus on Lake Baikal in Siberia and the Arrival of Small-Scale Pastoralism

**DOI:** 10.1038/s41598-017-02636-w

**Published:** 2017-05-24

**Authors:** Robert J. Losey, Andrea L. Waters-Rist, Tatiana Nomokonova, Artur A. Kharinskii

**Affiliations:** 1 Department of Anthropology, 13-8 Tory Building, University of Alberta, Edmonton AB, T6R 3H8 Canada; 2 Faculty of Archaeology, Leiden University, PO Box 9514, 2300 RA Leiden, The Netherlands; 3 Community, Culture and Global Studies, University of British Columbia Okanagan, 1147 Research Road, Kelowna, BC V1Y 1V7 Canada; 4 Irkutsk National Research Technical University, Lermontov St. 83, Irkutsk, 664074 Russian Federation

## Abstract

The spread of pastoralism in Asia is poorly understood, including how such processes affected northern forager populations. Lake Baikal’s western shore has a rich Holocene archaeological record that tracks these processes. The Early Bronze Age here is evidenced by numerous forager burials. The Early Iron Age (EIA) is thought to mark the arrival of pastoralists, but archaeological remains from this period have received little analysis. New radiocarbon dates for EIA human remains from 23 cemeteries indicate that no burials were created along this shore for ~900 years. This period, from ~3670 to 2760 cal. BP, spans from the end of the Early Bronze Age to the advent of the EIA. The burial gap may mark disruption of local foraging populations through incursions by non-local pastoralists. Radiocarbon dates on faunal remains indicate that domestic herd animals first appear around 3275 cal. BP, just prior to the first EIA human burials. Stable carbon and nitrogen isotope analysis of human remains and zooarchaeological data indicate that domestic fauna were minor dietary components for EIA people. Like preceding foragers, the EIA groups relied extensively on Baikal’s aquatic food sources, indicating that the scale of pastoralism during this period was relatively limited.

## Introduction

Pastoralism was critical to many major social and economic transitions in the prehistory of Eurasia, particularly in its steppe zones^1–5^. Mobile Early Iron Age pastoralist groups of the steppes had extensive and diverse impacts upon adjacent communities, with those affecting nearby literate empires and states being the best documented^6–8^. Far less well-understood are the interactions between animal-keeping groups and North Asia’s foraging societies, and the ideological and subsistence transformations that occurred when pastoralists or pastoralism moved into the forest-steppe and taiga regions adjacent to the steppe zone.

**Figure 1:**
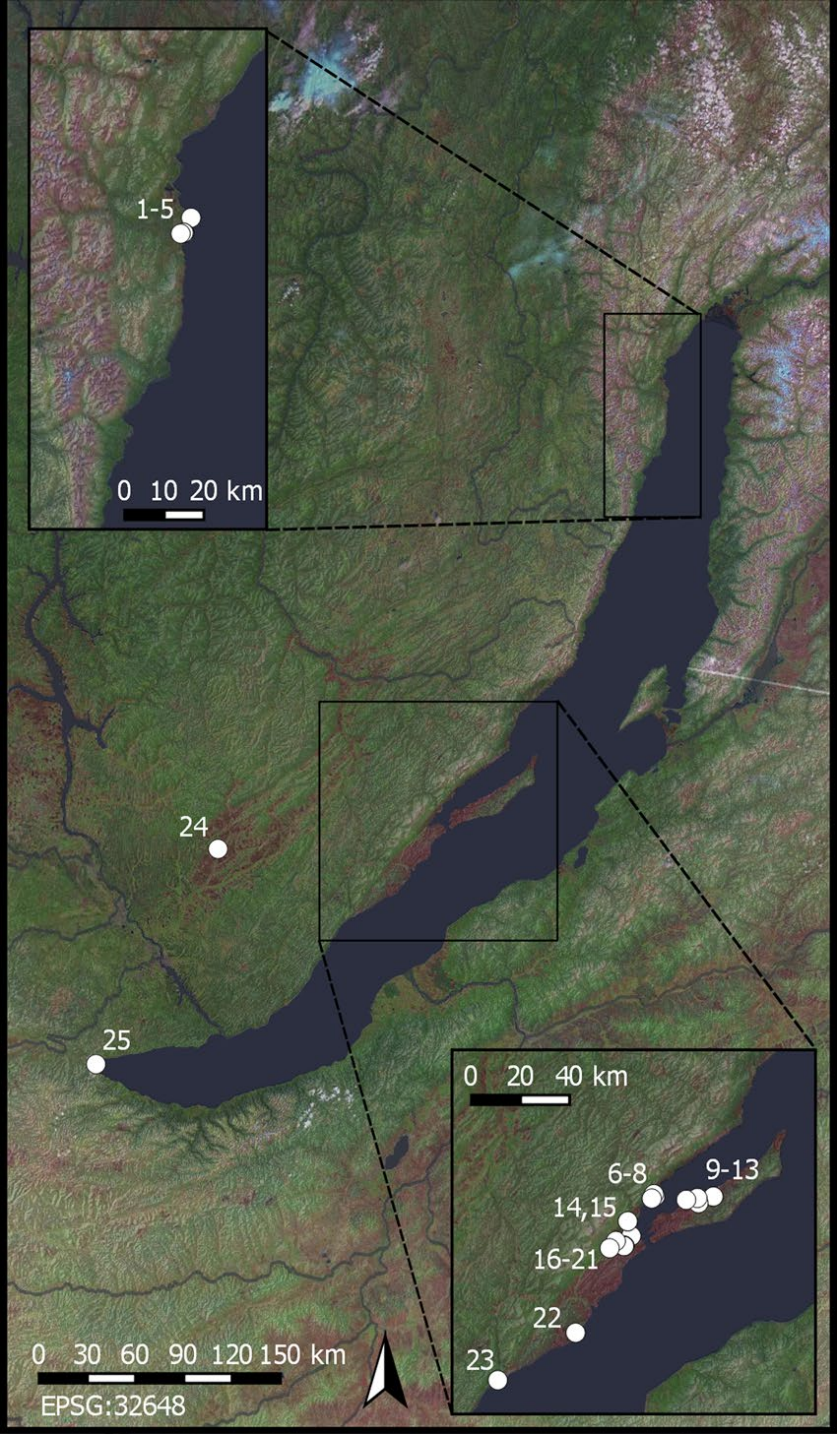
Map of the Lake Baikal region of the Russian Federation: (a) North Baikal; (b) Priol’khon’e. Archaeological sites analyzed in the study indicated: 1) Relka 1; 2) Baikal’skoe 7; 3) Baikal’skoe 27; 4) Baikal’skoe 31; 5) Krasnyi Iar; 6) Kurma 2; 7) Khadarta 2; 8) Tsagan-Nuge 2; 9) Khuzhirtui 1; 10) Elga 21; 11) Elga 7; 12) Khuzhir 2; 13) Khuzhir 4; 14) Khuzhir-Nuge 18; 15) Khuzhir Nuge 3; 16) Olzontei 16; 17) Kargarnai 1; 18) Olzontei 6; 19) Shara-Tagot; 20) Kurkut 4; 21) Olzontei 8; 22) Sagan-Zaba 2; 23) Bugul’deika 2; 24) Mankhai 3; 25) Shamanka 2. Landsat data courtesy of the U.S. Geological Survey. Landsat image tiles, which are open access files, were acquired through QGIS QuickMapServices^62^.

The western shore of Lake Baikal in Siberia is a compelling setting for exploring such transitions and processes (Fig. 1). This region is ecologically disjunct from the steppe areas of Mongolia to the south, Trans-Baikal to the east, and the Minusinsk Basin to the west, being separated from by them by mountain ranges, stretches of forest, or Lake Baikal itself. This region is one of the most aquatic-rich areas of interior North Asia, having abundant river and lake fish, as well as Baikal seals^9, 10^. Patches of steppe and forest steppe also are present along the central western shore of the lake in the Priol’khon’e region^11^. Most importantly, this region’s Middle Holocene hunter-gatherer archaeology is one of the most thoroughly studied in North Asia^12, 13^.

Hundreds of sets of Middle Holocene human skeletal remains from graves in Priol’khon’e, the Angara River Valley, and the Upper Lena have been repeatedly radiocarbon dated, and their carbon and nitrogen stable isotope values determined^14–17^. Radiocarbon dating of stratigraphically-associated deer and Baikal seal remains from habitation sites along the Priol’khon’e shoreline, in conjunction with paired human and terrestrial faunal bone from individual graves in various regions west of the lake, have all demonstrated major locally-specific freshwater reservoir effects (FRE) in the region’s aquatic food webs, rendering the radiocarbon ages of some human graves as much as 600 years too old^18–20^. Bayesian modeling of the radiocarbon dates for the region’s Middle Holocene cemeteries, including local corrections for the FREs, indicate that foragers began regularly burying their dead in these areas around 8300 cal. BP^15^. A gap occurs in this burial record between 7000 and 5600 cal. BP, which is followed by two sequential phases of forager interment, the latter persisting until ~3700 cal. BP^15^. The latter phase of forager burials is termed the Early Bronze Age (EBA). Stable carbon and nitrogen isotope data indicate that foragers in all of these regions and time periods had diets of mixed aquatic and terrestrial food types, with lake fish and Baikal seal being significant staples for some individuals buried in Priol’khon’e and on the lake’s southwest shore^14–17^.

Areas along the western shore of Lake Baikal also contain a rich Late Holocene archaeological record. Much of this derives from Early Iron Age pastoralists, including graves and other ritual features typologically assigned to several mortuary traditions or cultures^21, 22^. The best known is the Slab Grave (Plitochnaia) tradition, which is thought to mark the earliest settlement of the region by pastoralists^21, 22^. Slab graves are widespread across southern Trans-Baikal and much of eastern and northern Mongolia; this culture is almost exclusively known through its mortuary sites, as habitation sites of this period are rare^23, 24^. On the western shore of Baikal, graves assigned to the Slab Grave and other Early Iron Age mortuary traditions (Butuheiskoe, Elginskoe) sometimes hold iron weaponry and other metal items such as horse bits, as well as skeletal remains of domestic animals (horse, cattle, sheep, goat)^25–27^.

Despite the importance of the Early Iron Age in the region’s prehistory, little research has been dedicated to understanding the timing of its appearance, including how this relates to the chronology of local forager mortuary traditions, and how diets and subsistence practices of these two groups and periods compare. One limiting factor has been the lack of stable isotope data for the region’s Early Iron Age human remains, which would allow their radiocarbon dates to be corrected for the FREs and dietary patterns to be better understood. No systematic radiocarbon dating of these human remains has been made prior to this study. Equally important, two Late Holocene habitation sites along the western Baikal shoreline with extensively dated faunal assemblages are now available, providing additional data on local subsistence and dietary patterns^28, 29^.

This paper provides the first analyses of stable isotope values and AMS radiocarbon dates for human remains from the western shore of Lake Baikal post-dating the Early Bronze Age. We also integrate zooarchaeological data from two habitation sites, which provide additional insight on the relative importance of domestic ungulates and wild fauna in the diet and subsistence practices.

## Results

For this study, 47 AMS radiocarbon dates on adult human remains from graves typologically assigned to the Early Iron Age are available, including 11 from North Baikal, 34 from Priol’khon’e, and one each from the Kuda Valley and South Baikal; all have δ^13^C and δ^15^N values and come from a total of 23 cemeteries (Fig. 1; see Supplementary Tables 1 and 2. Six radiocarbon dates on domestic ungulate remains recovered from some of these cemeteries also are available (see Supplementary Table S3). These appear to be remains of sacrifices conducted around the time of human mortuary rites that were subsequently interred near or in the grave. The uncalibrated ages of the human burials range from ~2850 to 1985 BP (see Supplementary Table S2). In comparison, the Early Bronze Age forager burials in this region have uncalibrated ages ranging from ~4410 to 3410 BP^15^. The dates on domestic animal remains from the Early Iron Age cemeteries have uncalibrated ages ranging between ~2585 to 2360 BP, provisionally post-dating the earliest Early Iron Age burials by a few centuries (see Supplementary Table S3).

**Figure 2:**
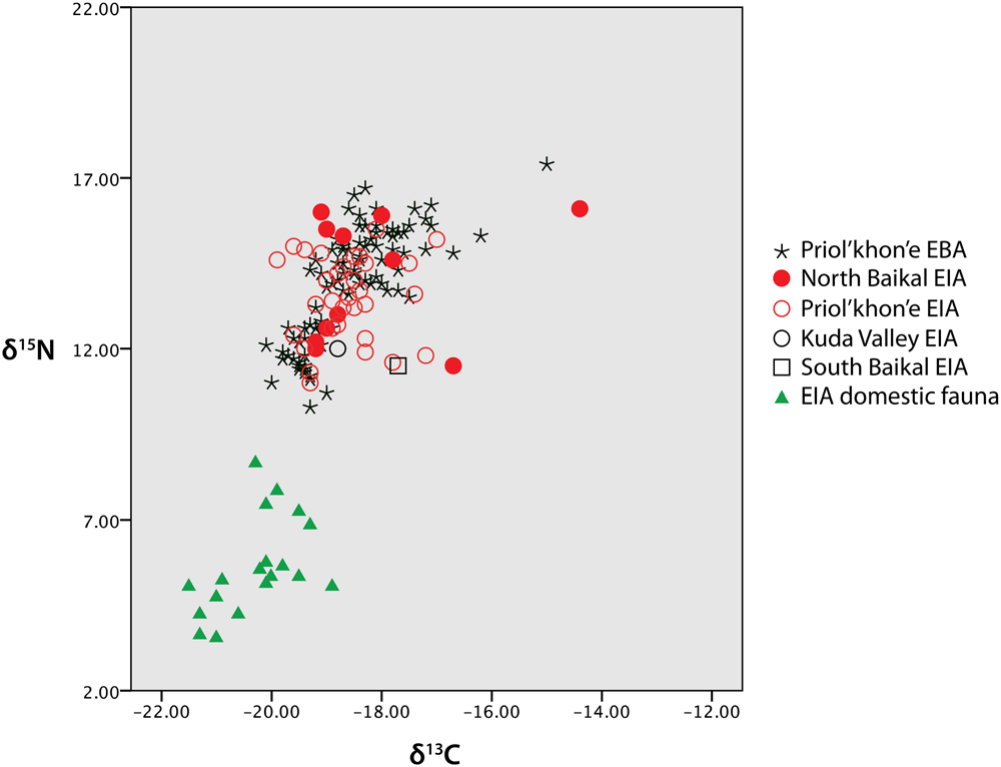
Stable isotope values for Early Bronze Age (EBA) and Early Iron Age (EIA) human bone collagen, and for EIA domestic fauna. Human samples are labeled by subregion and time period.

Stable isotope analysis show that the Early Iron Age human remains have a mean δ^13^C value of −18.5‰ (s.d. = 0.92) and δ^15^N value of 13.6‰ (s.d. = 1.39) (Fig. 2; see Supplementary Table S1). Remains from domestic fauna recovered from the Early Iron Age cemeteries, including 19 total horse, cattle, and caprine specimens, have δ^13^C values ranging from −21.3 to −18.9‰, and δ^15^N values from 3.6 to 7.9‰, with an overall average δ^13^C value of −20.3‰ (s.d. = 0.7) and δ^15^N value of 5.7‰ (s.d. = 1.4) (see Supplementary Table S1). The previously reported δ^15^N mean value for wild ungulates from this same region is 5.1‰ (s.d. = 1.0), and average δ^13^C value is −19.6‰ (s.d. = 0.8), both similar to those of the domestic fauna^17^.

Bone collagen δ^15^N values provide an indication of trophic level, with an expected enrichment of 3–6‰ along the food chain^30–32^. The mean δ^15^N value of the EIA human remains is 7.9‰ higher than the mean δ^15^N value of the domestic ungulates (13.6‰ versus 5.7‰, respectively), and 8.5‰ higher than that of the wild ungulates (5.1‰). The δ^15^N values of the EIA humans range from 11.0 to 15.9‰ (Supplementary Table S1.), meaning that the individual with the lowest δ^15^N values is 5.3‰, or about one trophic level, above the average for the domestic fauna. Even using the highest trophic level fractionation value of 6‰, these comparisons indicate that most EIA individuals were consuming protein from sources other than just domestic or wild ungulates. Previously reported mean δ^15^N values for Lake Baikal fish and seal are 11.5‰ (s.d. = 1.7) and 13.7‰ (s.d. = 1.1), respectively^17^. Both have mean δ^15^N values at least one full trophic level above those of local terrestrial ungulates, and consumption of these aquatic resources could easily account for the elevated δ^15^N values of the EIA humans observed in this study. Further to this point, the mean δ^15^N value for the EIA humans (13.6‰) is almost identical to that of the Baikal seal, which feeds exclusively on lake fish.

The δ^13^C values of human remains from the Baikal region are far less straightforward to interpret in regard to consumption of aquatic foods^19^. Fish from the lake have highly variable δ^13^C values, with bone collagen values ranging from −28.6 to −9.6‰^17^. Such variability occurs because of the extremes in basic productivity within the lake, which range from very negative δ^13^C values among the phytoplankton in its deeper sections, to far more positive values among the blue-green algae in its shallower sections^33, 34^. Fish can feed in sections of the lake dominated by both of these extremes over their lives, or feed largely in just one of them, resulting in highly variably δ^13^C values even within individual species^35^. Further, the higher δ^13^C values for fish from Lake Baikal overlap with those of C4 plants, adding yet more complication to interpretation of δ^13^C values. Millet, a C4 plant, has not been documented in the study area during the periods of interest here, but was identified at the Iron Age Ivolgin site near Ulan-Ude, ~100 east of Lake Baikal^36^, and thus was potentially consumed by the EIA individuals studied here. The other major aquatic food source, the Baikal seal, has a far narrower range of δ^13^C values than the fish, from −23.2 to −21.0‰ (mean = −22.1‰)^17^, reflecting its preferential diet of a subset of the lake’s deeper water fish. The δ^13^C values for the EIA humans are quite variable, ranging from −19.9 to −14.4‰, with a mean of 18.5‰. This range of variation is likely indicative of variable reliance on terrestrial fauna, Baikal seals, shallow or deep water feeding fish, and even C4 plants such as millet.

Perhaps most informative, the average Early Iron Age human isotope values are statistically indistinguishable from those of the EBA hunter-gatherer burials (n = 90) in Priol’khon’e (Fig. 2) (δ^13^C: t = −0.573, p = 0.568; δ^15^N: t = −0.367, p = 0.714)^17^. The isotope values of these hunter-gatherers have been interpreted as indicating diets of variable amounts of terrestrial mammals, fish, and Baikal seals^14, 17^; consumption of C4 plants such as millet has not been invoked in interpretations of these individuals’ diets. Further, the Early Iron Age humans have higher average δ^15^N values than EBA hunter-gatherers from both the Angara (n = 13; δ^15^N mean = 11.4‰, s.d. = 0.9) and Upper Lena rivers (n = 14; δ^15^N mean = 10.3‰, s.d. = 0.9)^17^. These forager groups are said to have had protein diets consisting mostly of freshwater fish and terrestrial mammals^17^. Together these comparisons provide additional indication that the Early Iron Age individuals analyzed here consumed a diet consisting of a mix of terrestrial and aquatic fauna.

Additional indications that the Late Holocene groups inhabiting the western shore of Baikal consumed terrestrial fauna and the lake’s seals and fish are the faunal remains recovered from two habitation sites, Bugul’deika 2 and Sagan-Zaba 2 (Fig. 1). Both are on the open shoreline of Baikal in Priol’khon’e and appear to have been occupied from late winter through early summer^28, 29^. The faunal remains from these habitation sites most likely provide a better overall picture of the relative dietary importance of types of animals in the diet (during the seasons of site occupation) than faunal remains from mortuary contexts, the latter being created through sacrifice and feasting associated with human mortuary rites, not daily practices.

**Figure 3:**
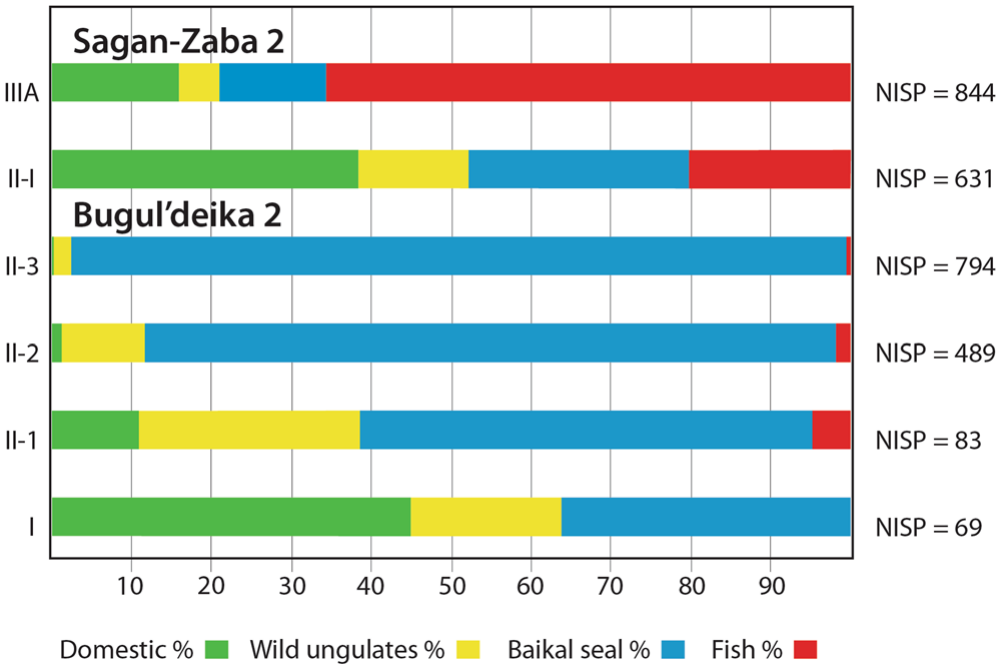
Identified faunal remains from the Sagan-Zaba 2 and Bugul’deika 2 habitation sites. Analytical unit designations are given on the left margin, and the numbers of identified specimen (NISP) values for each analytical unit are listed on the right margin.

Bugul’deika 2 has four components with remains of domestic ungulates, which represent intermittent site use from ~3100 cal. BP to the modern period^28^. The earliest component (II-3) produced a single domestic animal specimen, a horse bone, directly dated to ~2920 to 2780 cal. BP. Baikal seal remains constitute 97.1% of the faunal assemblage from component II-3, indicating a clear focus on sealing at the site during this period (Fig. 3). One cattle specimen from component II-2 was directly dated to 2680 to 2350 cal. BP, and this layer also produced remains of horse and caprines^28^. Domestic ungulate remains become more relatively abundant starting in II-2 and through the latter two components (II-1, and I), but Baikal seals still account for at least 35% of the identified specimens in all three analytical units. Fish remains are rare in all Bugul’deika 2 components, likely due to the rare use of sieves during excavation.

Sagan-Zaba 2, which was consistently sieved during excavation, has two components with remains of domestic ungulates, representing intermittent occupation of the site area from ~1970 and 940 cal. BP^29^. In the earlier of the two components (IIIA), domesticate remains account for only 16.0% of the total faunal assemblage, and wild ungulates just 5.1%, while fish and Baikal seal constitute a total of 78.9% (Fig. 3)^29^. The faunal assemblage from the latest component (II-I) shows an increase in the relative importance of domestic ungulates, which form 38.4% of the total remains, however fish and seal still make up 47.9% of the component, and wild ungulates 13.8%. The earliest directly dated domestic ungulate remains at Sagan-Zaba 2 are from a horse in component IIIA, which dates to 1880 to 1730 cal. BP^29^.

Given the stable isotope and zooarchaeological indications of human consumption of both terrestrial and aquatic fauna during the Late Holocene, the Early Iron Age human radiocarbon dates should be just as biased by the region’s freshwater reservoir effects as are those of its previous forager inhabitants, who had similarly structured diets. For this study, the Early Iron Age human dates were corrected using the same methods developed for the earlier forager burials (see Supplementary Tables S2 and S4). The corrections to the uncalibrated dates ranged from 110 to 500 years (Tables S2 and S4). The 47 Early Iron Age dates, and 91 recently published dates for Early Bronze Age burials from this same region^15^ were then modeled to estimate the start and end dates for the two phases of burial, and the amount of time that elapsed between the end of the Early Bronze Age burials and the advent of those assigned to the Early Iron Age (Table 1, see Supplementary Tables 5–8. The trapezium distribution model indicates that the Early Iron Age burials first appeared between 2760 to 2485 cal. BP. The uniform distribution model estimates a similar age range for the advent of these burials, at 2775 to 2515 BP. Both models indicate a clear temporal gap in human burials in this region of over 900 years, with the estimated end of the EBA burials occurring by ~3630 BP.

**Table 1:** Modeled radiocarbon ages for human remains and domestic faunal remains.

	Trapezium Model		Uniform Distribution Model	
	Modeled Cal BP age (95.4%)	Modeled BC/AD age (95.4%)	Modeled Cal BP age (95.4%)	Modeled BC/AD age (95.4%)
Human remains				
Early Bronze Age end	3800 to 3670 BP	1850 to 1720 BC	3760 to 3630 BP	1815 to 1680 BC
Early Iron Age start	2760 to 2485 BP	810 to 535 BC	2775 to 2515 BP	825 to 565 BC
Early Iron Age end	2080 to 1710 BP	130 BC to 245 AD	1905 to 1690 BP	50 to 260 AD
Span EBA end to EIA start	960 to 1270 years	960 to 1270 years	na	na
Faunal remains				
Domestic fauna start	3275 to 2605 BP	1325 to 655 BC	3180 to 2780 BP	1230 to 830 BC
Domestic fauna end	2220 to 1440 BP	275 BC to 510 AD	1890 to 1475 BP	60 to 475 AD

Date ranges are given at 95% confidence levels.

The radiocarbon dates on domestic fauna remains from the Early Iron Age cemeteries and habitation site components (n = 10) were also modeled as a single phase, using both trapezium and uniform distribution models (Table 1, see Supplementary Table S9). The mean δ^15^N value for these samples is 5.8‰ (s.d. = 1.3), indicating these animals were not consuming aquatic fauna and that their radiocarbon dates are not biased by the region’s FREs. The modeled starting age ranges for these dates predate and somewhat overlap with the modeled starting date ranges for the Early Iron Age human burials, but in neither model do they overlap in age with the Early Bronze Age dates. In other words, domestic herd animals were present in the region slightly before Early Iron Age burials first appear. Caution is warranted, as the total number of dated domestic fauna is quite small in comparison to the number of dated human remains.

Finally, to assess if there were chronological trends in the Early Iron Age stable isotope data, the human burials’ mean modeled radiocarbon ages were plotted against their δ^15^N and δ^13^C values (see Supplementary Fig. S1). These analyses showed no significant correlation in either isotope ratio by age, indicating no statistically significant trends in the diets of these individuals through time.

## Discussion

The multi-century temporal gap in human burials between the end of the Early Bronze Age and start of Early Iron Age was entirely unrecognized prior to this study. This gap is unlikely to be a product of sampling or research effort. First, the region’s burials have been extensively excavated and radiocarbon dated, with hundreds of dates on human remains and the faunal bone artifacts associated with them now available^12, 15, 16, 19, 20^. This includes radiocarbon dating of Early Bronze Age graves from all three sub-regions west of Lake Baikal (Angara-Southwest Baikal, Priol’khon’e, Upper Lena), with none of these dating to the gap period. Second, while Okladnikov^37^ originally identified several Bronze Age mortuary traditions in this region based on grave typology, subsequent radiocarbon dating of graves matching his types along the western Baikal shore has shown that all date within the Early Bronze Age period as it is now defined^15, 38^. The Late Bronze Age is currently unrepresented in the region’s mortuary remains. Third, the western shore of Lake Baikal has been repeatedly surveyed for archaeological sites since the 1940s, and the Priol’khon’e region in particular has experienced significant development for tourism over the last two decades or so, resulting in numerous surveys and test excavations. In other words, it seems unlikely that the gap in burials can be attributed to lack of effort to locate sites.

It is presently unclear why this temporal gap in burials occurs, but it may relate to broader regional environmental and cultural changes. We are unaware of any significant correlation between environmental change and the end of the Early Bronze Age burials in this region. One remaining possibility is that groups from neighboring areas disrupted the foragers living along Baikal’s west shore through brief incursions, resulting in population dispersal or even abandonment of some areas. Conceivably, such groups could have been pastoralists from areas such as Trans-Baikal to the east or Mongolia to the south. Similar shifts or differences in demography (related in part to environmental changes) have been linked to the lack of interments in this region in previous research^13^, and may also explain the lack of burials dating to the gap period identified here. However, there is currently no evidence the end of the Early Bronze Age was due to permanent settlement of western shore of Baikal by pastoral groups. Specifically, even the earliest date on domestic ungulates from the western shore of the lake (Table S3) post-dates the end of the Early Bronze Age by centuries, and the modeled date range for the domestic fauna is at least 400 years after the modeled end range of these earlier burials (Table 1). The directly dated domestic animals described here, however, have a modeled age range mostly predating the Early Iron burials. This suggests animal keeping may have begun in this region a few centuries before the first Iron Age graves appear. Notably, the number of well dated habitation sites from this period and region is very low (there are only two), as is the number of dates on domestic fauna. Clearly, confirmation of this pattern with additional data is warranted.

The advent of Early Iron Age burials along the western shore appears to correlate with an episode of marked local environmental change. Analysis of pollen from a lake core in the Priol’khon’e region indicates that this area experienced a very high rate of vegetation turnover from 2750 to 2480 cal. BP, marking the onset of drier continental conditions^39^. Such conditions may have been more suitable for animal-keeping groups, but this hypothesis requires additional research. Conversely, steppe biomes were clearly present in this region prior to this period of abrupt change during the Middle Holocene^39^, suggesting that environments potentially suitable for maintaining herd animals were available here long before such animals are present in the archaeological record. Regardless, the advent of Early Iron Age burials along the western shore of Baikal likely represents the initiation of permanent settlement of the region by groups keeping herd animals. The radiocarbon dates indicate that Iron Age graves were constructed along parts of the western shore for many centuries, and remains of domestic animals were present a few centuries earlier. Genetic studies and additional radiocarbon dating (and assessment for local FREs) of Iron Age graves in adjacent regions, such as Trans-Baikal, are needed to determine if this represents colonization of the region by outside groups, the adoption of animal-keeping by local people, or some combination of both processes.

The average extent of reliance on domestic herd animals as food sources appears to have been relatively minor among the Early Iron Age individuals studied here. The stable isotope and zooarchaeological data both indicate substantial reliance on the region’s aquatic fauna, including lake fish and Baikal seal. The stable isotope data provide no indication that this pattern in food consumption changed over the course of the Early Iron Age. The zooarchaeological record suggests a slow increase in the relative abundance of domestic animals over the entire Late Holocene, not an abrupt transition to heavily reliance on these animals during the Early Iron Age. The low level of pastoralism along the western shore is also supported by local pollen and spore records, which provide no evidence for extensive herding in the region until well into the AD 1800s^39–41^.

Overall, while the Early Iron Age groups of the western Baikal shore clearly kept domestic herd animals, they had diets reminiscent of the region’s earlier Bronze Age foraging populations. Animal-keeping during this period was small-scale, and was carried out in conjunction with a significant amount of both hunting and fishing, as is seen in this region today^10^. The mixed pastoral-foraging practices indicated by our data are in many ways not surprising, as ancient groups in many areas of Central Asia clearly had diets consisting of a mix of wild and domestic animals, which in some areas included aquatic fauna^42–47^. In other words, these groups often had broader diets than the term ‘pastoral’ implies^42^—people were keeping herd animals, but were often not wholly reliant upon them as dietary items.

Movements of animal-keeping populations into the boreal forest region of Siberia likely were major transformative processes. For example, such northward migrations of pastoral societies have long have been linked to a number of important developments, ranging from the origins of reindeer husbandry^48^, to the arrival of Turkic-speaking groups and their horses in the Arctic^49^, to the emergence of highly stratified settled societies along the Pacific Coast of the Russian Far East^50^. Clearly these episodes of dispersal and cultural change affecting vast areas of North Asia are worthy of additional intensive study, including more detailed analyses of the interactions between resident groups and newcomers. Reliable chronologies are fundamental for such studies, and in many cases refining these will require adjustments in radiocarbon dates for old carbon effects, whether they are due to the use of marine or freshwater foods. We hope this study offers inspiration for such research in the near future.

## Methods

### Stable Isotope Analysis

Standard δ^13^C and δ^15^N extraction and preparation procedures for bone collagen were followed^51, 52^. All bone samples were cleaned manually and then placed in distilled water (dH_2_O) and repeatedly washed ultrasonically until all sediment was removed. Dry bone samples were demineralized in a dilute 1% hydrochloric acid (HCl) solution, changed every 24–48 hours until complete. Collagen psuedomorphs were then rinsed in dH_2_O until neutrality was reached and then transferred into a 0.1 M solution of sodium hydroxide (NaOH) for 20 hours to remove humic acids. Finally, samples were again rinsed in dH_2_O to neutrality and then freeze-dried. After freeze drying, collagen yields were calculated with the total dry bone weight expressed as a percentage of the starting weight.

Measurement of δ^13^C and δ^15^N occurred on a continuous flow Delta V plus isotope ratio mass spectrometer, paired with a Thermo-Scientific Flash 2000 organic elemental analyser at Vrije Universiteit, Amsterdam, Department of Earth Sciences, Cluster Geology and Geochemistry. Three criteria are used to assess the preservation of extracted collagen: atomic C/N ratio, which should fall between 2.9–3.6^52–55^, collagen yield, which should minimally be higher than 1.0%^53, 55, 56^, and the percent of C and N in the extracted collagen, which based on collagen from modern animals should be between ~35–47% for C and ~11–17.3% for N^51, 54^. The precision of the mass spectrometer based on repeat measurements is 0.1‰ for δ^13^C and 0.2‰ for δ^15^N. The precision of percentages C and N in collagen, measured via the elemental analyzer, are +/−5.0%.

### Reservoir Effect Correction

The Early Iron Age human dates were corrected for FREs in two ways. The first utilized a generalized regression equation for the region constructed using paired human-terrestrial mammal dates from graves in Priol’khon’e, South Baikal/Angara River, and the Upper Lena River combined (see Supplementary Table S2) ^19^. The model takes into account the δ^13^C and δ^15^N values of each dated individual in making the corrections. This equation is preferred because it allows all burial dates to be adjusted in the same way, and leaves open the possibility that the individuals inhabited several of the areas west of Lake Baikal, incorporating carbon from all of them into their skeletal remains. This method is the preferred one, and was used in calculating the modeled calibrated age ranges presented in the text. The second method employed local equations where possible (e.g., the Priol’khon’e and South Baikal/Angara regression equations for burials from those respective regions)^19^, and used the general equation when local equations were lacking (e.g., for North Baikal burials and the individual from the Kuda Valley) (see Supplementary Table S5). This method allowed some buried individuals to be treated as local to their respective regions.

### Radiocarbon Dating, Calibration, and Modeling

All radiocarbon dates were calibrated and modeled in Oxcal 4.2.4 using the IntCal-13 dataset^57–59^. Calibrated age ranges are reported in years BP to be consistent with previous publications on the region’s hunter-gatherer mortuary sites; BC/AD dates are provided in Table 1 for clarification, as Late Holocene sites in Central Asia are commonly described in this format. The FRE-corrected Early Bronze Age human dates^15^, and the Early Iron Age human dates, all corrected for FREs using the general regression equation for Cis-Baikal^19^, were entered into two different types of phase models in Oxcal. The first is the trapezium distribution model, which allows for potential overlap in the two phases (EBA and EIA), and gradual changes in deposition rates (see Supplementary Table S5)^60, 61^. The second is the uniform distribution model, which assumes that the dates are uniformly distributed within each phase, and that abrupt transitions occurred between phases (see Supplementary Table S7). Alternative results also are provided for both models when using EIA dates that are corrected (where possible) using the locally specific FRE corrections (see Supplementary Tables S6 and S8). Note that these results differed very little from those obtained in the preferred models presented in the text. The domestic fauna radiocarbon dates were entered into single phase uniform and trapezium models without correction for FREs given their low δ^15^N values (see Supplementary Table S9). In all models, the corrected dates are also presented with their mean, standard deviation, and median ages in years cal. BP. Dates ranges provided in Table 1 are shown at 95% confidence levels.

### Archaeology and Zooarchaeology

Grave numbers and typological assignments were translated as presented in the primary Russian-language literature. Faunal remains were identified using a comparative collection at Irkutsk National Research Technical University during this study. These remains are quantified by number of identified specimens (NISP), and the identified specimens are those assigned to order level or below.

## Electronic supplementary material

Supplementary Figures and Tables
