# Supplementary material for: A Second Mortuary Hiatus on Lake Baikal in Siberia and the Arrival of Small-Scale Pastoralism
Source: Sci Rep. 2017 May 24;7:2319. doi: 10.1038/s41598-017-02636-w (PMC5443801; doi:10.1038/s41598-017-02636-w)
Supplement: Supplementary file 1 — Supplementary Figures and Tables [file 41598_2017_2636_MOESM1_ESM.pdf]

SUPPLEMENTARY FILES:

A Second Mortuary Hiatus on Lake Baikal in Siberia and the Arrival of Small-Scale Pastoralism

Robert J. Losey[a*], Andrea L. Waters-Rist[b], Tatiana Nomokonova[c], Artur A. Kharinskii[d]

a. Department of Anthropology, 13-8 Tory Building, University of Alberta, Edmonton AB, T6R 3H8, Canada

b. Faculty of Archaeology, Leiden University, PO Box 9514, 2300 RA Leiden, The Netherlands

c. Community, Culture and Global Studies, University of British Columbia Okanagan 1147 Research Road, Kelowna, BC, V1Y 1V7, Canada

d. Irkutsk National Research Technical University, Lermontov St. 83, Irkutsk, 664074, Russian Federation

*Corresponding author: robert.losey@ualberta.ca

Figure S1. Iron Age human stable isotope values plotted against mean modeled radiocarbon ages. Uncalibrated ages were corrected for the freshwater reservoir effect using the general regression for Cis-Baikal (19). Modeled mean ages were calculated using trapezium distribution model (58). Pearson's R^2 value and regression slope equation are provided on each plot. a) $\delta^{13}C$ values by mean modeled age; b) $\delta^{15}N$ values by mean modeled age.

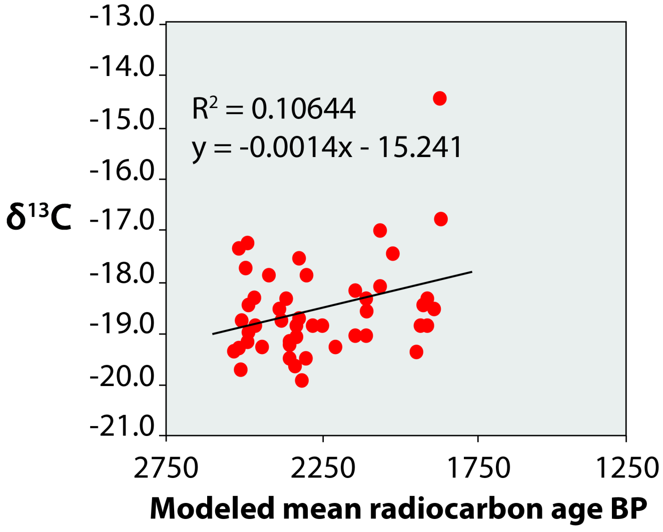

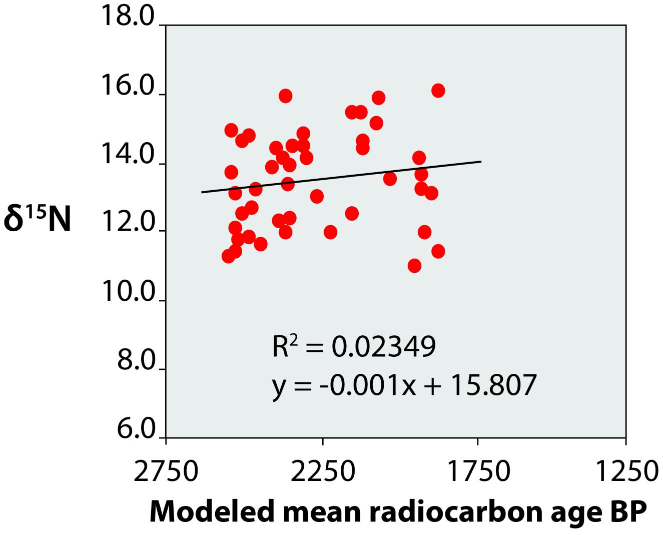

Table S1. Stable carbon and nitrogen isotope values for Early Iron Age human and fauna remains.

Site	Grave/Feature	Material	%N by weight	%C by weight	Atomic C/N ratio	Collagen yield	$\delta^{13}C$ (‰ VPDB)	$\delta^{15}N$ (‰ AIR)	Typological Period	Reference
**North Baikal**										
Relka 1	Grave 5	Human	16.1	44.7	3.2	3.4	**-18.0**	**15.9**	Plitochnaia	61
Relka 1	Grave 1	Human	16.0	45.1	3.3	7.0	**-19.0**	**12.6**	Plitochnaia	61
Baikalskoe 7	Grave 2	Human	15.7	45.1	3.4	7.0	**-19.0**	**15.5**	Butuheiskoe	*This study*
Baikal'skoe 7	Grave 1	Human	n/a	n.a	3.4	n/a	**-16.7**	**11.5**	Butuheiskoe	*This study*
Baikal'skoe 27	Complex 8	Human	15.6	45.5	3.4	13.5	**-19.2**	**12.0**	Butuheiskoe	*This study*
Baikal'skoe 27	Complex 12	Human	n/a	n./a	3.4	n/a	**-19.2**	**12.2**	Butuheiskoe	*This study*
Baikal'skoe 31	Grave 8	Human	15.7	45.4	3.4	7.7	**-17.8**	**14.6**	Butuheiskoe	*This study*
Baikal'skoe 31	Complex 4	Human	n/a	n/a	3.5	n/a	**-18.8**	**13.0**	Butuheiskoe	*This study*
Baikal'skoe 31	Grave 5	Human	15.5	43.9	3.3	5.9	**-14.4**	**16.1**	Butuheiskoe	*This study*
Baikal'skoe 31	Complex 9	Human	15.5	44.1	3.3	5.7	**-19.1**	**16.0**	Butuheiskoe	*This study*
Krasnyi Iar I	Grave 3	Human	16.0	44.4	3.2	15.0	**-18.7**	**14.4**	Butuheiskoe	*This study*
**Little Sea**										
Khuzhirtui 1	Complex 1	Human	15.2	42.4	3.3	1.7	**-18.8**	**14.2**	Plitochnaia	61
Khuzhir-Nuge 18	Grave 3	Human	16.5	45.9	3.2	14.5	**-17.8**	**11.6**	Plitochnaia	61
Khuzhir-Nuge III	Grave 9	Human	16.0	44.4	3.2	15.0	**-18.7**	**14.4**	Plitochnaia	61
Kurma 2	Grave 5	Human	16.4	45.2	3.2	18.9	**-18.3**	**14.5**	Butuheiskoe	61
Olzontei 16	Grave 1	Human	16.9	46.5	3.2	19.5	**-18.7**	**14.4**	Plitochnaia	61
Olzontei 6	Grave 4	Human	16.5	45.8	3.2	12.2	**-18.3**	**12.3**	Plitochnaia	61
Olzontei 6	Grave 2-1	Human	16.3	45.5	3.3	13.8	**-19.6**	**12.4**	Plitochnaia	61
Elga 21	Grave 1	Human	16.2	45.5	3.3	17.2	**-19.6**	**15.0**	Plitochnaia	61
Elga 21	Complex 4	Human	16.3	45.3	3.2	11.6	**-19.1**	**14.8**	Plitochnaia	61
Elga 21	Grave 5	Human	16.7	46.4	3.2	19.6	**-18.3**	**11.9**	Butuheiskoe	61
Elga 21	Complex 3	Human	16.7	47.0	3.3	12.5	**-18.8**	**12.7**	Plitochnaia	61
Khadarta 2	Grave 5	Human	16.2	45.3	3.3	12.8	**-17.5**	**14.5**	Butuheiskoe	61
Kargarnai 1	Grave 7	Human	17.0	48.2	3.3	10.6	**-19.4**	**12.0**	Plitochnaia	61
Kargarnai 1	Complex 1	Human	17.0	47.4	3.2	14.6	**-17.3**	**13.7**	Plitochnaia	61
Kargarnai 1	Complex 4	Human	16.5	46.0	3.3	11.0	**-19.2**	**13.3**	Plitochnaia	61
Kargarnai 1	Grave 5	Human	15.7	43.7	3.2	11.4	**-18.7**	**13.2**	Plitochnaia	61
Elga VII	Grave 2	Human	n/a	n/a	3.4	n/a	**-18.3**	**13.3**	Elginskoe	61
Elga VII	Grave 3	Human	16.7	47.6	3.3	13.0	**-19.3**	**11.0**	Elginskoe	61
Shara-Tagot	Grave 2, 1985	Human	17.0	46.6	3.2	15.3	**-18.9**	**12.6**	Plitochnaia	61
Shara-Tagot	Grave 1, 1985	Human	16.4	45.3	3.2	4.9	**-18.9**	**13.4**	Plitochnaia	61
Kurkut IV	Grave 20	Human	14.9	42.0	3.3	2.1	**-19.9**	**14.6**	Plitochnaia	61
Elga 21 1996	Grave 2	Human	16.9	47.1	3.2	19.5	**-19.3**	**11.3**	Plitochnaia	*This study*

Khuzhir 2	Feature 2	Human	16.4	45.9	3.3	14.7	**-18.4**	**14.7**	Plitochnaia	*This study*
Khuzhir 4	Feature 9	Human	16.5	46.0	3.3	11.6	**-19.0**	**14.0**	Plitochnaia	*This study*
Khuzhir 4	Grave 11	Human	17.4	48.0	3.2	21.6	**-19.4**	**14.9**	Plitochnaia	*This study*
Olzontei 8	Complex 1	Human	16.9	14.0	3.2	20.5	**-17.2**	**11.8**	Plitochnaia	*This study*
Tsagan-Khushun IIB	Grave 20	Human	17.3	47.7	3.2	17.8	**-18.5**	**13.2**	Elginskoe	*This study*
Tsagan-Khushun IIA	Grave 23	Human	16.4	45.2	3.2	16.5	**-18.1**	**15.5**	Butuheiskoe	*This study*
Tsagan-Khushun IIB	Complex 16	Human	16.5	45.1	3.2	21.5	**-18.4**	**13.7**	Elginskoe	*This study*
Tsagan-Khushun IIB	Grave 4	Human	16.9	46.7	3.2	19.3	**-18.8**	**14.2**	Elginskoe	*This study*
Tsagan-Khushun IIA	Grave 19	Human	17.1	46.9	3.2	19.8	**-18.5**	**14.0**	Elginskoe	*This study*
Tsagan-Khushun IIA	Grave 31	Human	17.2	47.1	3.2	17.5	**-18.5**	**14.7**	Butuheiskoe	*This study*
Tsagan-Khushun IIB	Grave 27	Human	n/a	n/a	3.3	n/a	**-17.4**	**13.6**	Elginskoe	*This study*
Tsagan-Khushun IIA	Grave 6	Human	16.6	45.9	3.2	17.9	**-17.0**	**15.2**	Elginskoe	*This study*
**Kuda Valley**										
Mankhai 3	Grave 11	Human	17.3	47.7	3.2	20.3	**-18.8**	**12.0**	Plitochnaia	61
**South Baikal**										
Shamanka 2	Grave 106	Human	na	43.2	3.1	20.9	**-17.7**	**11.5**	Early Iron Age	15
**Fauna Samples**										
Khuzhir 4	feature 11	*Bos*	17.2	47.1	3.2	21.1	**5.8**	**-20.1**	Plitochnaia	*This study*
Khuzhir 4	feature 8	*Bos*	17.1	48.2	3.3	19.4	**7.5**	**-20.1**	Plitochnaia	*This study*
Khuzhir 4	12278-2	*Equus*	17.5	48.7	3.2	17.4	**4.3**	**-20.6**	Plitochnaia	*This study*
Elga XXI	complex 3	*Equus*	15.3	44.8	3.4	5.2	**5.1**	**-21.5**	Plitochnaia	61
Olzontei VI	feature 1	*Equus*	16.5	46.4	3.3	14.4	**5.3**	**-20.9**	Plitochnaia	61
Khuzhir 4	feature 10 1227	*Equus*	17.1	47.6	3.2	15.7	**4.3**	**-21.3**	Plitochnaia	*This study*
Olzontei VI	feature 2	*Equus*	15.8	44.7	3.3	6.0	**3.6**	**-21.0**	Plitochnaia	61
Relka I	grave 3 skull 4	*Equus*	16.5	46.1	3.3	13.7	**4.8**	**-21.0**	Plitochnaia	61
Relka I	grave 3 skull 5	*Equus*	16.4	45.5	3.2	13.1	**3.7**	**-21.3**	Plitochnaia	61
Elga XXI	grave 5	*Equus*	16.5	46.0	3.3	8.2	**7.9**	**-19.9**	Butuheiskoe	*This study*
Khuzhir 4	feature 1a	*Ovis*	16.1	45.3	3.3	16.8	**7.3**	**-19.5**	Plitochnaia	*This study*
Olzontei VI	grave 2 skull 2	*Ovis*	16.4	45.4	3.2	16.1	**5.4**	**-20.0**	Plitochnaia	61
Olzontei VI	grave 2 skull 5	*Ovis*	16.3	45.2	3.2	14.3	**5.4**	**-19.5**	Plitochnaia	61
Olzontei VI	grave 2 skull 6	*Ovis*	16.4	45.8	3.3	14.2	**5.7**	**-19.8**	Plitochnaia	61
Elga XXI	grave 4	*Ovis*	16.0	45.5	3.3	4.8	**5.6**	**-20.2**	Plitochnaia	*This study*
Kurma II	grave 5	*Ovis*	16.4	45.6	3.2	14.5	**6.9**	**-19.3**	Plitochnaia	61
Olzontei VI	feature 1 skull 6	*Capra*	16.9	47.0	3.2	14.1	**5.1**	**-18.9**	Plitochnaia	*This study*
Khuzhir 4	feature 1a	*Capra*	16.7	47.1	3.3	19.1	**8.7**	**-20.3**	Plitochnaia	*This study*
Elga XXI	bone concentrat	Caprinae	15.8	45.5	3.4	4.2	**5.2**	**-20.1**	Plitochnaia	*This study*

Samples below were excluded from calculations and figures because two are juveniles, and one is a wild animal.

Khuzhir 4	12282-19	*Capreolus*	18.0	48.1	3.2	20.1	**7.4**	**-21.0**	Plitochnaia	*This study*
Olzontei VI	feature 1 skull 5	*Equus* (nev	16.3	45.5	3.3	9.9	**6.0**	**-20.2**	Plitochnaia	*This study*
Olzontei VI	feature 1	*Equus* (juv	17.0	46.7	3.2	17.5	**3.7**	**-21.1**	Plitochnaia	*This study*

Table S2. Radiocarbon dates on Early Iron Age human remains. Dates corrected for the freshwater reservoir effect using the general regression equation for Cis-Baikal (19).

Site	Grave/Feature	Material	Lab Number	14C age BP	Age error	Corrected age	Corrected σ	Adjusted age difference	Typological Period	Reference
**North Baikal**										
Relka 1	Grave 5	Human	Ua-49659	2527	34	2042	92	485	Plitochnaia	61
Relka 1	Grave 1	Human	Ua-49660	2357	33	2125	92	232	Plitochnaia	61
Baikalskoe 7	Grave 2	Human	Ua-49630	2548	32	2094	91	455	Butuheiskoe	*This study*
Baikal'skoe 7	Grave 1	Human	Ua-49628	1986	30	1838	91	148	Butuheiskoe	*This study*
Baikal'skoe 27	Complex 8	Human	Ua-49622	2382	31	2196	91	186	Butuheiskoe	*This study*
Baikal'skoe 27	Complex 12	Human	Ua-49623	2725	33	2523	92	202	Butuheiskoe	*This study*
Baikal'skoe 31	Grave 8	Human	Ua-49624	2669	31	2283	91	386	Butuheiskoe	*This study*
Baikal'skoe 31	Complex 4	Human	Ua-49625	2502	30	2239	91	263	Butuheiskoe	*This study*
Baikal'skoe 31	Grave 5	Human	Ua-49626	2335	30	1835	91	500	Butuheiskoe	*This study*
Baikal'skoe 31	Complex 9	Human	Ua-49627	2814	34	2321	92	493	Butuheiskoe	*This study*
Krasnyi Iar I	Grave 3	Human	Ua-49670	2750	32	2311	91	439	Butuheiskoe	*This study*
**Little Sea**										
Khuzhirtui 1	Complex 1	Human	Ua-49648	2621	49	2266	99	355	Plitochnaia	61
Khuzhir-Nuge 18	Grave 3	Human	Ua-49649	2527	33	2371	92	156	Plitochnaia	61
Khuzhir-Nuge III	Grave 9	Human	Ua-49650	2705	37	2335	93	370	Plitochnaia	61
Kurma 2	Grave 5	Human	Ua-49653	2470	32	2092	91	378	Butuheiskoe	61
Olzontei 16	Grave 1	Human	Ua-49655	2674	35	2304	92	370	Plitochnaia	61
Olzontei 6	Grave 4	Human	Ua-49656	2541	33	2332	92	209	Plitochnaia	61
Olzontei 6	Grave 2-1	Human	Ua-49657	2524	33	2307	92	217	Plitochnaia	61
Elga 21	Grave 1	Human	Ua-49633	2952	32	2536	91	416	Plitochnaia	61
Elga 21	Complex 4	Human	Ua-49634	2827	31	2426	91	401	Plitochnaia	61
Elga 21	Grave 5	Human	Ua-49635	2607	32	2428	91	179	Butuheiskoe	61
Elga 21	Complex 3	Human	Ua-49631	2651	31	2411	91	240	Plitochnaia	61
Khadarta 2	Grave 5	Human	Ua-49643	2681	37	2303	93	378	Butuheiskoe	61
Kargarnai 1	Grave 7	Human	Ua-49638	2507	30	2321	91	186	Plitochnaia	61
Kargarnai 1	Complex 1	Human	Ua-49639	2833	33	2532	92	301	Plitochnaia	61
Kargarnai 1	Complex 4	Human	Ua-49641	2678	31	2392	91	286	Plitochnaia	61
Kargarnai 1	Grave 5	Human	Ua-49642	2760	31	2482	91	278	Plitochnaia	61
Elga VII	Grave 2	Human	Ua-49637	2187	30	1901	91	286	Elginskoe	61
Elga VII	Grave 3	Human	Ua-49636	2047	20	1937	88	110	Elginskoe	61
Shara-Tagot	Grave 2, 1985	Human	Ua-49662	2680	33	2448	92	232	Plitochnaia	61

Shara-Tagot	Grave 1, 1985	Human	Ua-49663	2605	33	2311	92	294	Plitochnaia	61
Kurkut IV	Grave 20	Human	Ua-49652	2680	35	2294	92	386	Plitochnaia	61
Elga 21 1996	Grave 2	Human	Ua-49632	2706	30	2573	91	133	Plitochnaia	*This study*
Khuzhir 2	Feature 2	Human	Ua-49645	2849	46	2456	97	393	Plitochnaia	*This study*
Khuzhir 4	Feature 9	Human	Ua-49646	2646	34	2306	92	340	Plitochnaia	*This study*
Khuzhir 4	Grave 11	Human	Ua-49647	2694	35	2285	92	409	Plitochnaia	*This study*
Olzontei 8	Complex 1	Human	Ua-49658	2641	34	2470	92	171	Plitochnaia	*This study*
Tsagan-Khushun IIB	Grave 20	Human	Ua-49664	2153	32	1875	91	278	Elginskoe	*This study*
Tsagan-Khushun IIA	Grave 23	Human	Ua-49665	2580	32	2126	91	455	Butuheiskoe	*This study*
Tsagan-Khushun IIB	Complex 16	Human	Ua-49666	2219	36	1902	93	317	Elginskoe	*This study*
Tsagan-Khushun IIB	Grave 4	Human	Ua-49667	2273	30	1918	91	355	Elginskoe	*This study*
Tsagan-Khushun IIA	Grave 19	Human	Ua-49668	2683	37	2343	93	340	Elginskoe	*This study*
Tsagan-Khushun IIA	Grave 31	Human	Ua-49669	2481	34	2088	92	393	Butuheiskoe	*This study*
Tsagan-Khushun IIB	Grave 27	Human	Ua-49671	2321	32	2012	91	309	Elginskoe	*This study*
Tsagan-Khushun IIA	Grave 6	Human	Ua-49672	2482	33	2050	92	432	Elginskoe	*This study*
**Kuda Valley**										
Mankhai 3	Grave 11	Human	Ua-49654	2080	32	1894	91	186	Plitochnaia	61
**South Baikal**										
Shamanka 2	Grave 106	Human	OxA-21498	2633	27	2485	90	148	Early Iron Age	15

Table S3. Radiocarbon dates on Early Iron Age domestic fauna remains from habitation sites and cemeteries.

Site	Grave/Feature	Material	Lab Number	Sample Number	14C age BP	Age error	$\delta^{13}C$ (‰VPDB)	δ15N (‰AIR)	Atomic C/N ratio	Reference
Habitation sites										
Bugul'deika II	Trench 5 Layer II-3	*Equus*	OxA23939	E 2010.020	2751	26	-20.5	3.8	3.3	28
Bugul'deika II	Trench 5 Layer II-2	*Bos*	OxA23994	E 2010.012	2406	27	-20.6	6.2	3.4	28
Bugul'deika II	Trench 5 Layer I	*Caprinae*	OxA23989	E 2010.005	2008	28	-19.8	8.5	3.3	28
Sagan-Zaba II	Trench 4C layer IIIA	*Equus*	OxA22389	t4cf895	1882	26	-21.1	3.2	3.2	29
Cemeteries										
Relka 1	Grave 3 skull 4	*Equus*	Ua-49674	2014-Relka1/3-4-1	2357	33	-20,6	4.2	3,0	61
Olzontei 6	Feature 1	*Ovis*	Ua-49673	2014-Olzontei6/1-1	2496	32	-19,3	5.9	2,8	61
Elga 21	Grave 5	*Caprinae*	Ua-49677	2014-Elga21/5-2	2436	35	-19,2	5.8	2,8	61
Kargarnai 1	Grave 7	*Caprinae*	Ua-49676	2014-Karg1/7-2	2584	34	-20,0	5.6	2,7	61
Khuzhir 4	Feature 9	*Caprinae*	Ua-49675	2014-Khuzh4/9-2	2380	33	-21.2	6.9	2.9	*This study*
Shamanka 2	Feature 84	*Equus*	Ua-53580	2016-Sham-Fea84	2414	28	-20.5	5.2	3.2	*This study*

Table S4. Radiocarbon dates on Early Iron Age human remains. Dates corrected for the freshwater reservoir effect using subregion-specific regression equations for Cis-Baikal (19). GE is the general regression equation, LS is the Little Sea regression, and SB/AN is the South Baikal/Angara regression.

Site	Grave/Feature	Material	Lab Number	14C age BP	Age error	Correction Regression Equation	Corrected age	Corrected σ	Adjusted age difference	Typological Period	Reference
**North Baikal**											
Relka 1	Grave 5	Human	Ua-49659	2527	34	GE	2042	92	485	Plitochnaia	61
Relka 1	Grave 1	Human	Ua-49660	2357	33	GE	2125	92	232	Plitochnaia	61
Baikalskoe 7	Grave 2	Human	Ua-49630	2548	32	GE	2094	91	455	Butuheiskoe	*This study*
Baikal'skoe 7	Grave 1	Human	Ua-49628	1986	30	GE	1838	91	148	Butuheiskoe	*This study*
Baikal'skoe 27	Complex 8	Human	Ua-49622	2382	31	GE	2196	91	186	Butuheiskoe	*This study*
Baikal'skoe 27	Complex 12	Human	Ua-49623	2725	33	GE	2523	92	202	Butuheiskoe	*This study*
Baikal'skoe 31	Grave 8	Human	Ua-49624	2669	31	GE	2283	91	386	Butuheiskoe	*This study*
Baikal'skoe 31	Complex 4	Human	Ua-49625	2502	30	GE	2239	91	263	Butuheiskoe	*This study*
Baikal'skoe 31	Grave 5	Human	Ua-49626	2335	30	GE	1835	91	500	Butuheiskoe	*This study*
Baikal'skoe 31	Complex 9	Human	Ua-49627	2814	34	GE	2321	92	493	Butuheiskoe	*This study*
Krasnyi Iar I	Grave 3	Human	Ua-49670	2750	32	GE	2311	91	439	Butuheiskoe	*This study*
**Little Sea**											
Khuzhirtui 1	Complex 1	Human	Ua-49648	2621	49	LS	2239	71	382	Plitochnaia	61
Khuzhir-Nuge 18	Grave 3	Human	Ua-49649	2527	33	LS	2518	61	9	Plitochnaia	61
Khuzhir-Nuge III	Grave 9	Human	Ua-49650	2705	37	LS	2316	64	389	Plitochnaia	61
Kurma 2	Grave 5	Human	Ua-49653	2470	32	LS	2122	61	348	Butuheiskoe	61
Olzontei 16	Grave 1	Human	Ua-49655	2674	35	LS	2285	63	389	Plitochnaia	61
Olzontei 6	Grave 4	Human	Ua-49656	2541	33	LS	2402	61	139	Plitochnaia	61
Olzontei 6	Grave 2-1	Human	Ua-49657	2524	33	LS	2212	61	312	Plitochnaia	61
Elga 21	Grave 1	Human	Ua-49633	2952	32	LS	2393	61	559	Plitochnaia	61
Elga 21	Complex 4	Human	Ua-49634	2827	31	LS	2350	60	477	Plitochnaia	61
Elga 21	Grave 5	Human	Ua-49635	2607	32	LS	2506	61	101	Butuheiskoe	61
Elga 21	Complex 3	Human	Ua-49631	2651	31	LS	2411	60	240	Plitochnaia	61
Khadarta 2	Grave 5	Human	Ua-49643	2681	37	LS	2434	64	247	Butuheiskoe	61
Kargarnai 1	Grave 7	Human	Ua-49638	2507	30	LS	2259	60	248	Plitochnaia	61
Kargarnai 1	Complex 1	Human	Ua-49639	2833	33	LS	2542	61	291	Plitochnaia	61
Kargarnai 1	Complex 4	Human	Ua-49641	2678	31	LS	2331	60	347	Plitochnaia	61
Kargarnai 1	Grave 5	Human	Ua-49642	2760	31	LS	2485	60	275	Plitochnaia	61
Elga VII	Grave 2	Human	Ua-49637	2187	30	LS	1953	60	234	Elginskoe	61

Elga VII	Grave 3	Human	Ua-49636	2047	20	LS	1906	56	141	Elginskoe	61
Shara-Tagot	Grave 2, 1985	Human	Ua-49662	2680	33	LS	2437	61	243	Plitochnaia	61
Shara-Tagot	Grave 1, 1985	Human	Ua-49663	2605	33	LS	2286	61	319	Plitochnaia	61
Kurkut IV	Grave 20	Human	Ua-49652	2680	35	LS	2122	63	558	Plitochnaia	61
Elga 21 1996	Grave 2	Human	Ua-49632	2706	30	LS	2537	60	169	Plitochnaia	*This study*
Khuzhir 2	Feature 2	Human	Ua-49645	2849	46	LS	2469	69	380	Plitochnaia	*This study*
Khuzhir 4	Feature 9	Human	Ua-49646	2646	34	LS	2258	62	388	Plitochnaia	*This study*
Khuzhir 4	Grave 11	Human	Ua-49647	2694	35	LS	2170	63	524	Plitochnaia	*This study*
Olzontei 8	Complex 1	Human	Ua-49658	2641	34	LS	2688	62	-47	Plitochnaia	*This study*
Tsagan-Khushun IIB	Grave 20	Human	Ua-49664	2153	32	LS	1904	61	249	Elginskoe	*This study*
Tsagan-Khushun IIA	Grave 23	Human	Ua-49665	2580	32	LS	2162	61	418	Butuheiskoe	*This study*
Tsagan-Khushun IIB	Complex 16	Human	Ua-49666	2219	36	LS	1935	63	284	Elginskoe	*This study*
Tsagan-Khushun IIB	Grave 4	Human	Ua-49667	2273	30	LS	1891	60	382	Elginskoe	*This study*
Tsagan-Khushun IIA	Grave 19	Human	Ua-49668	2683	37	LS	2357	64	326	Elginskoe	*This study*
Tsagan-Khushun IIA	Grave 31	Human	Ua-49669	2481	34	LS	2089	62	392	Butuheiskoe	*This study*
Tsagan-Khushun IIB	Grave 27	Human	Ua-49671	2321	32	LS	2172	61	149	Elginskoe	*This study*
Tsagan-Khushun IIA	Grave 6	Human	Ua-49672	2482	33	LS	2231	61	251	Elginskoe	*This study*
**Kuda Valley**											
Mankhai 3	Grave 11	Human	Ua-49654	2080	32	GE	1894	91	186	Plitochnaia	61
**South Baikal**											
Shamanka 2	Grave 106	Human	OxA-21498	2633	27	SB/AN	2583	70	50	Early Iron Ag	15

Table S5. Oxcal (57, 58) output for Early Bronze Age (15) and Early Iron Age human radiocarbon dates using the trapezium distribution model (60) and adjusted for the FRE using the general regression model for Cis-Baikal (19).

	**Unmodelled age cal. BP**						**Modelled age cal. BP**					
	**from**	**to**	**%**	**μ date**	**σ**	**Median date**	**from**	**to**	**%**	**μ HPD date**	**μ HPD σ**	**Median date**
Sequence Cis-Baikal EBA n=91												
Boundary EBA Start							4633	4367	95.4	4496	70	4492
Start							5110	4893	95.4	4994	57	4987
Transition							697	1274	95.4	996	150	1004
End							4251	3760	95.4	3998	133	3988
Phase												
R_Date SHM_1975.001	5276	4861	95.4	5014	110	4991	4995	4852	95.4	4909	38	4900
R_Date SMS_1986.009	5271	4827	95.5	4956	100	4937	4989	4710	95.4	4880	57	4879
R_Date UID_1991.042	5029	4570	95.4	4777	112	4784	4883	4530	95.4	4721	95	4718
R_Date GO2_1996.003	4959	4528	95.4	4737	102	4735	4860	4528	95.4	4696	92	4696
R_Date GO2_1996.004	4855	4455	95.4	4693	96	4697	4841	4449	95.4	4661	95	4660
R_Date GO2_1995.002	4845	4447	95.4	4674	100	4677	4831	4442	95.4	4641	99	4638
R_Date OBK_1976.003	4826	4530	95.4	4678	89	4678	4824	4455	95.4	4652	87	4646
R_Date KUR_2002.009	4815	4425	95.4	4606	110	4588	4808	4421	95.4	4575	100	4560
R_Date OBK_1971.013	4806	4358	95.4	4513	91	4497	4786	4298	95.4	4495	78	4485
R_Date MAK_1992.018	4781	4259	95.4	4458	82	4464	4575	4256	95.4	4445	78	4451
R_Date KUR_2003.025	4529	4159	95.4	4374	94	4374	4525	4159	95.4	4360	91	4360
R_Date KUR_2003.026	4524	4160	95.4	4362	89	4363	4520	4158	95.4	4350	87	4350
R_Date MAK_1992.019	4516	4161	95.4	4346	76	4348	4514	4159	95.4	4337	76	4337
R_Date OBK_1971.003.02	4514	4159	95.4	4339	76	4341	4509	4157	95.4	4330	75	4331
R_Date BAD_1920.000	4520	4099	95.4	4320	102	4323	4515	4095	95.3	4305	100	4308
R_Date UBE_1957.002	4517	4100	95.4	4315	97	4319	4513	4095	95.4	4301	96	4305
R_Date OBK_1971.001.02	4421	4185	95.4	4327	59	4331	4421	4183	95.4	4322	59	4325
R_Date MAK_1992.013.00	4436	4155	95.4	4318	76	4323	4427	4154	95.4	4310	76	4314
R_Date KUR_2003.018	4435	4094	95.4	4289	90	4293	4427	4092	95.4	4278	90	4281
R_Date KHA_2010.011	4434	4093	95.4	4288	90	4292	4426	4091	95.4	4277	90	4280
R_Date OBK_1971.001.03	4419	4101	95.4	4279	83	4280	4416	4100	95.4	4269	83	4269
R_Date MKV_1973.001	4418	4100	95.4	4274	84	4275	4416	4097	95.4	4265	84	4265
R_Date OBK_1971.007	4417	4100	95.4	4274	84	4274	4415	4098	95.4	4264	84	4264
R_Date KHA_2010.015	4421	4090	95.4	4266	94	4268	4418	4090	95.4	4255	94	4255
R_Date KUR_2002.007.01	4425	4084	95.4	4260	98	4261	4421	4014	95.4	4248	98	4248
R_Date KUL_1977.000	4420	4084	95.4	4248	97	4247	4417	4015	95.4	4236	96	4234

R_Date KUR_2002.013	4419	4014	95.4	4243	98	4240	4416	4014	95.4	4231	97	4228
R_Date KUR_2002.014	4417	4013	95.4	4236	97	4233	4414	4009	95.4	4225	97	4221
R_Date OBK_1971.001.01	4407	4091	95.4	4228	82	4221	4402	4089	95.4	4220	80	4212
R_Date KUR_2002.010	4415	4010	95.4	4228	97	4223	4412	4006	95.4	4216	97	4211
R_Date KUR_2002.003	4413	3999	95.4	4214	102	4210	4410	3994	95.4	4202	101	4199
R_Date KUR_2002.012	4411	3994	95.4	4203	103	4200	4407	3991	95.4	4191	101	4188
R_Date KUR_2002.005	4410	3992	95.4	4198	102	4196	4405	3990	95.4	4187	100	4184
R_Date OBK_1971.004.01	4406	3999	95.4	4195	90	4192	4400	3993	95.4	4186	88	4183
R_Date UID_1991.039	4413	3985	95.4	4193	115	4192	4406	3984	95.4	4179	113	4177
R_Date KUR_2002.007.02	4409	3990	95.4	4192	104	4190	4405	3986	95.4	4180	102	4177
R_Date SMS_1986.012	4408	3991	95.4	4190	102	4188	4405	3987	95.5	4179	100	4176
R_Date OBK_1971.003.01	4406	3994	95.4	4189	91	4186	4400	3990	95.4	4180	89	4177
R_Date KUR_2003.017	4406	3987	95.4	4180	100	4178	4398	3985	95.4	4170	98	4168
R_Date SMS_1987.033	4405	3987	95.4	4179	100	4176	4388	3984	95.3	4168	98	4166
R_Date KHA_2010.005	4405	3983	95.3	4169	102	4166	4395	3980	95.4	4158	100	4156
R_Date K14_2000.077	4401	3980	95.4	4156	97	4153	4386	3976	95.4	4147	94	4145
R_Date KUR_2002.001	4405	3976	95.3	4153	101	4149	4396	3935	95.4	4142	99	4139
R_Date OBK_1971.004.02	4350	3980	95.4	4149	88	4146	4296	3979	95.4	4141	86	4139
R_Date KUR_2003.019	4380	3929	95.3	4129	97	4126	4347	3927	95.4	4120	94	4118
R_Date KUR_2002.006	4385	3926	95.4	4127	101	4123	4349	3922	95.4	4117	97	4115
R_Date SHM_1973.001	4346	3913	95.4	4111	99	4109	4289	3913	95.4	4102	95	4101
R_Date KHA_2010.009	4294	3910	95.4	4107	97	4106	4286	3913	95.4	4099	94	4098
R_Date OBK_1971.014.02	4245	3929	95.4	4098	82	4099	4243	3930	95.4	4092	80	4094
R_Date KUR_2002.015	4289	3910	95.4	4101	96	4099	4285	3905	95.4	4093	93	4092
R_Date SMS_1986.013	4287	3904	95.4	4095	95	4093	4283	3902	95.4	4088	92	4086
R_Date OBK_1971.014.01	4240	3929	95.4	4089	80	4090	4239	3929	95.4	4084	79	4085
R_Date KHA_2010.008	4238	3895	95.4	4062	90	4058	4234	3895	95.4	4056	88	4051
R_Date MNZ_1974.004.01	4225	3899	95.4	4049	75	4045	4225	3897	95.4	4045	73	4041
R_Date UIA_1977.005	4217	3888	95.4	4030	73	4030	4156	3885	95.4	4027	72	4027
R_Date K14_1998.037.01	4228	3865	95.4	4031	86	4030	4225	3859	95.4	4026	84	4025
R_Date KUR_2002.016	4218	3850	95.4	4017	83	4016	4155	3849	95.4	4013	81	4012
R_Date KUR_2002.004	4150	3849	95.4	4001	80	3999	4149	3852	95.4	3998	78	3995
R_Date KHA_2010.012	4150	3842	95.4	3998	84	3995	4149	3844	95.4	3995	82	3991
R_Date SHM_1973.002	4150	3837	95.4	3989	85	3984	4148	3838	95.4	3987	83	3981
R_Date UIA_surface	4138	3837	95.4	3967	74	3961	4091	3837	95.4	3966	72	3959
R_Date K14_1999.057.02	4148	3829	95.4	3966	85	3960	4146	3829	95.4	3965	81	3959
R_Date K14_2001.087	4148	3778	95.4	3963	86	3958	4147	3827	95.4	3963	82	3956
R_Date SHM_1972.002	4084	3842	95.4	3957	66	3949	4084	3843	95.4	3956	64	3948

R_Date UKH_1930.000	4089	3735	95.4	3938	74	3933	4086	3830	95.4	3938	71	3933
R_Date KHA_2003.003	4090	3726	95.5	3931	83	3928	4090	3736	95.4	3934	78	3928
R_Date OBK_1971.005	4085	3730	95.4	3922	76	3919	4085	3773	95.4	3925	72	3920
R_Date MNZ_1974.001	4080	3724	95.4	3903	76	3902	4081	3732	95.5	3908	70	3904
R_Date SMS_1987.021	4084	3720	95.4	3902	87	3901	4083	3730	95.4	3909	79	3905
R_Date SHA_2000.009	4086	3712	95.4	3900	99	3900	4083	3732	95.4	3911	88	3906
R_Date SHM_1972.001.01	4080	3702	95.4	3883	90	3885	4079	3726	95.4	3894	81	3891
R_Date K14_1998.036.01	4080	3702	95.4	3883	90	3885	4079	3726	95.4	3894	81	3891
R_Date SHA_2008.103.02	4084	3694	95.4	3878	102	3879	4082	3723	95.4	3895	90	3891
R_Date SHM_1973.004	4079	3697	95.4	3873	90	3875	4076	3723	95.4	3886	80	3884
R_Date KHA_2010.007	4065	3649	95.4	3844	88	3848	4065	3714	95.4	3863	77	3862
R_Date K14_1999.045	3980	3645	95.4	3821	84	3824	3979	3711	95.4	3845	73	3844
R_Date BO1_1971.002	3971	3693	95.4	3818	74	3823	3970	3718	95.4	3837	65	3839
R_Date K14_1998.037.02	3975	3649	95.4	3818	80	3821	3975	3716	95.4	3840	70	3841
R_Date OBK_1971.010	3967	3651	95.4	3812	72	3814	3966	3716	95.4	3832	63	3834
R_Date SHA_2008.107	3984	3637	95.4	3812	92	3810	3985	3699	95.4	3844	77	3840
R_Date BO2_1971.002	3878	3650	95.4	3776	52	3775	3881	3710	95.4	3795	45	3795
R_Date K14_1999.049	3964	3634	95.4	3780	79	3779	3965	3697	95.4	3816	64	3813
R_Date BO1_1971.001	3900	3641	95.4	3775	67	3774	3910	3697	95.4	3804	55	3804
R_Date GLZ_1887.006	3971	3608	95.4	3778	91	3776	3972	3697	95.4	3823	71	3818
R_Date KHA_2010.006	3920	3612	95.4	3767	78	3767	3960	3693	95.4	3808	61	3806
R_Date SHA_2008.109	3965	3580	95.4	3765	93	3764	3970	3693	95.4	3817	70	3813
R_Date SHA_2008.103.01	3958	3584	95.4	3760	90	3760	3968	3690	95.4	3813	67	3809
R_Date BO2_1971.003	3872	3614	95.4	3748	67	3750	3886	3692	95.4	3790	49	3794
R_Date SHM_1973.003.01	3890	3590	95.4	3747	78	3748	3911	3685	95.4	3797	56	3799
R_Date UID_1994.048	3887	3496	95.4	3705	93	3701	3909	3665	95.4	3792	58	3796
R_Date SHA_2008.111	3841	3478	95.4	3670	96	3666	3893	3661	95.4	3784	56	3792
Span EBA dated events							1102	1312	95.4	1201	53	1195
Interval EBA							614	922	95.4	761	82	754
Boundary EBA End							3800	3670	95.4	3735	32	3736
Start							3921	3686	95.4	3791	60	3783
Transition							0	296	95.4	112	91	90
End							3771	3571	95.4	3679	51	3686
Sequence Cis-Baikal IA n=47												
Boundary IA Start							2760	2485	95.4	2622	73	2620
Start							2881	2515	95.4	2701	94	2702
Transition							0	469	95.4	160	147	118
End							2750	2321	95.4	2542	112	2546

Phase												
R_Date Ua-49659	2307	1819	95.4	2024	120	2016	2308	1832	95.4	2047	116	2038
R_Date Ua-49660	2333	1901	95.4	2120	118	2114	2336	1931	95.4	2133	115	2127
R_Date Ua-49630	2315	1882	95.4	2086	119	2078	2315	1898	95.4	2101	116	2093
R_Date Ua-49628	1970	1558	95.4	1766	107	1768	2043	1689	95.4	1858	86	1857
R_Date Ua-49622	2359	1948	95.4	2192	113	2198	2360	1952	95.4	2198	107	2205
R_Date Ua-49623	2765	2355	95.4	2582	118	2583	2694	2350	95.4	2510	93	2508
R_Date Ua-49624	2698	2042	95.4	2303	143	2291	2650	2043	95.3	2291	123	2286
R_Date Ua-49625	2485	1994	95.4	2239	122	2233	2456	2003	95.4	2238	110	2236
R_Date Ua-49626	1970	1556	95.4	1762	106	1765	2043	1688	95.4	1856	85	1855
R_Date Ua-49627	2705	2132	95.4	2375	156	2357	2667	2125	95.4	2348	132	2344
R_Date Ua-49670	2705	2119	95.4	2356	154	2339	2672	2116	95.4	2333	131	2331
R_Date Ua-49648	2696	1998	95.3	2282	148	2264	2610	1999	95.4	2272	128	2263
R_Date Ua-49649	2724	2159	95.4	2460	146	2448	2680	2158	95.4	2418	124	2415
R_Date Ua-49650	2709	2150	95.4	2402	157	2389	2672	2144	95.4	2369	133	2366
R_Date Ua-49653	2315	1880	95.4	2084	119	2075	2315	1896	95.4	2100	116	2091
R_Date Ua-49655	2705	2070	95.3	2343	153	2328	2672	2069	95.4	2322	130	2321
R_Date Ua-49656	2707	2149	95.4	2396	156	2382	2675	2142	95.4	2365	132	2360
R_Date Ua-49657	2705	2116	95.4	2348	154	2333	2671	2112	95.4	2327	131	2325
R_Date Ua-49633	2778	2357	95.4	2591	119	2591	2696	2351	95.4	2515	92	2513
R_Date Ua-49634	2744	2326	95.4	2519	126	2513	2705	2212	95.4	2467	106	2458
R_Date Ua-49635	2744	2329	95.4	2520	125	2515	2704	2310	95.4	2469	106	2459
R_Date Ua-49631	2744	2212	95.4	2506	130	2496	2700	2185	95.3	2457	110	2448
R_Date Ua-49643	2705	2069	95.4	2342	154	2327	2671	2067	95.3	2321	131	2320
R_Date Ua-49638	2705	2133	95.4	2375	156	2356	2670	2125	95.4	2348	132	2344
R_Date Ua-49639	2775	2356	95.4	2588	119	2588	2700	2351	95.4	2513	93	2512
R_Date Ua-49641	2738	2184	95.4	2487	137	2474	2693	2178	95.4	2441	116	2434
R_Date Ua-49642	2745	2355	95.4	2556	117	2559	2691	2345	95.4	2496	97	2491
R_Date Ua-49637	2060	1611	95.4	1839	112	1841	2114	1727	95.4	1905	95	1898
R_Date Ua-49636	2119	1633	95.4	1885	111	1885	2125	1745	95.4	1935	98	1927
R_Date Ua-49662	2743	2344	95.4	2534	122	2534	2701	2327	95.4	2480	102	2471
R_Date Ua-49663	2705	2119	95.4	2356	155	2340	2671	2116	95.4	2333	131	2331
R_Date Ua-49652	2701	2062	95.4	2324	150	2312	2660	2058	95.4	2307	128	2307
R_Date Ua-49632	2845	2365	95.4	2627	126	2627	2710	2355	95.4	2526	91	2525
R_Date Ua-49645	2750	2340	95.4	2538	124	2540	2704	2323	95.4	2481	103	2474
R_Date Ua-49646	2705	2115	95.4	2347	154	2332	2673	2071	95.4	2325	130	2324
R_Date Ua-49647	2699	2044	95.4	2308	146	2296	2653	2045	95.3	2294	125	2291
R_Date Ua-49658	2745	2351	95.4	2548	119	2551	2695	2340	95.4	2490	99	2483

R_Date Ua-49664	2002	1569	95.4	1807	109	1809	2104	1709	95.4	1884	91	1878
R_Date Ua-49665	2333	1903	95.4	2122	117	2116	2335	1931	95.4	2134	114	2128
R_Date Ua-49666	2101	1611	95.4	1841	115	1842	2115	1729	95.4	1908	97	1901
R_Date Ua-49667	2110	1623	95.4	1861	114	1861	2120	1735	95.4	1920	98	1913
R_Date Ua-49668	2712	2153	95.4	2416	156	2404	2671	2149	95.4	2381	132	2379
R_Date Ua-49669	2315	1876	95.4	2079	120	2071	2315	1894	95.4	2096	117	2087
R_Date Ua-49671	2300	1734	95.4	1986	118	1978	2304	1818	95.4	2014	112	2003
R_Date Ua-49672	2307	1822	95.4	2034	121	2026	2309	1863	95.4	2056	116	2046
R_Date Ua-49654	2055	1606	95.4	1830	111	1832	2111	1722	95.4	1899	94	1893
R_Date OxA-21498	2745	2356	95.4	2557	116	2561	2691	2345	95.4	2498	96	2493
Span IA dated events							668	1038	95.4	858	93	861
Interval IA							458	1002	95.4	742	145	749
Boundary IA End							2079	1707	95.4	1879	99	1866
Start							2482	1728	95.4	2068	229	2023
Transition							0	878	95.4	377	293	338
End							1872	1492	95.4	1691	99	1700
Difference Gap							961	1268	95.4	1113	80	1115

Table S6. Oxcal (57, 58) output for Early Bronze Age (15) and Early Iron Age human radiocarbon dates using the trapezium distribution model (60) and adjusted for the FRE using the subregion-specific regression models for Cis-Baikal (19).

	**Unmodelled age cal. BP**						**Modelled age cal. BP**					
	**from**	**to**	**%**	**µ date**	**σ**	**Median date**	**from**	**to**	**%**	**µ HPD date**	**µ HPD σ**	**Median date**
Sequence Cis-Baikal EBA n=91												
Boundary EBA Start							4634	4365	95.4	4495	71	4491
Start							5110	4892	95.4	4994	57	4987
Transition							694	1277	95.4	997	150	1006
End							4255	3760	95.4	3997	134	3986
Phase												
R_Date SHM_1975.001	5276	4861	95.4	5014	110	4991	4995	4852	95.4	4909	38	4900
R_Date SMS_1986.009	5271	4827	95.5	4956	100	4937	4989	4710	95.4	4880	56	4879
R_Date UID_1991.042	5029	4570	95.4	4777	112	4784	4881	4530	95.4	4721	95	4718
R_Date GO2_1996.003	4959	4528	95.4	4737	102	4735	4861	4528	95.4	4696	92	4695
R_Date GO2_1996.004	4855	4455	95.4	4693	96	4697	4840	4449	95.4	4661	94	4660
R_Date GO2_1995.002	4845	4447	95.4	4674	100	4677	4831	4443	95.4	4642	99	4638
R_Date OBK_1976.003	4826	4530	95.4	4678	89	4678	4824	4455	95.4	4652	87	4646
R_Date KUR_2002.009	4815	4425	95.4	4606	110	4588	4808	4420	95.4	4575	100	4560
R_Date OBK_1971.013	4806	4358	95.4	4513	91	4497	4787	4298	95.3	4495	78	4486
R_Date MAK_1992.018	4781	4259	95.4	4458	82	4464	4574	4256	95.4	4445	78	4451
R_Date KUR_2003.025	4529	4159	95.4	4374	94	4374	4525	4159	95.4	4360	91	4360
R_Date KUR_2003.026	4524	4160	95.4	4362	89	4363	4520	4159	95.4	4350	87	4350
R_Date MAK_1992.019	4516	4161	95.4	4346	76	4348	4513	4159	95.5	4337	76	4337
R_Date OBK_1971.003.02	4514	4159	95.4	4339	76	4341	4508	4158	95.4	4330	75	4331
R_Date BAD_1920.000	4520	4099	95.4	4320	102	4323	4516	4096	95.4	4305	100	4309
R_Date UBE_1957.002	4517	4100	95.4	4315	97	4319	4513	4095	95.4	4301	96	4305
R_Date OBK_1971.001.02	4421	4185	95.4	4327	59	4331	4421	4183	95.4	4322	59	4325
R_Date MAK_1992.013.00	4436	4155	95.4	4318	76	4323	4427	4155	95.4	4310	76	4314
R_Date KUR_2003.018	4435	4094	95.4	4289	90	4293	4428	4092	95.4	4278	90	4281
R_Date KHA_2010.011	4434	4093	95.4	4288	90	4292	4427	4091	95.4	4277	91	4280
R_Date OBK_1971.001.03	4419	4101	95.4	4279	83	4280	4416	4100	95.4	4269	83	4269
R_Date MKV_1973.001	4418	4100	95.4	4274	84	4275	4416	4097	95.4	4265	84	4264
R_Date OBK_1971.007	4417	4100	95.4	4274	84	4274	4415	4098	95.4	4265	84	4264
R_Date KHA_2010.015	4421	4090	95.4	4266	94	4268	4418	4090	95.4	4254	94	4254
R_Date KUR_2002.007.01	4425	4084	95.4	4260	98	4261	4422	4016	95.4	4248	98	4248
R_Date KUL_1977.000	4420	4084	95.4	4248	97	4247	4417	4014	95.4	4237	96	4234
R_Date KUR_2002.013	4419	4014	95.4	4243	98	4240	4416	4013	95.4	4231	97	4228

R_Date KUR_2002.014	4417	4013	95.4	4236	97	4233	4415	4010	95.4	4225	97	4220
R_Date OBK_1971.001.01	4407	4091	95.4	4228	82	4221	4403	4090	95.4	4220	80	4212
R_Date KUR_2002.010	4415	4010	95.4	4228	97	4223	4413	4006	95.4	4216	96	4211
R_Date KUR_2002.003	4413	3999	95.4	4214	102	4210	4410	3993	95.4	4202	101	4198
R_Date KUR_2002.012	4411	3994	95.4	4203	103	4200	4407	3991	95.4	4191	101	4188
R_Date KUR_2002.005	4410	3992	95.4	4198	102	4196	4404	3990	95.4	4186	100	4184
R_Date OBK_1971.004.01	4406	3999	95.4	4195	90	4192	4400	3995	95.5	4186	88	4183
R_Date UID_1991.039	4413	3985	95.4	4193	115	4192	4406	3984	95.4	4179	113	4177
R_Date KUR_2002.007.02	4409	3990	95.4	4192	104	4190	4405	3987	95.4	4180	102	4178
R_Date SMS_1986.012	4408	3991	95.4	4190	102	4188	4404	3987	95.4	4179	100	4176
R_Date OBK_1971.003.01	4406	3994	95.4	4189	91	4186	4398	3990	95.4	4179	89	4177
R_Date KUR_2003.017	4406	3987	95.4	4180	100	4178	4396	3984	95.5	4169	98	4167
R_Date SMS_1987.033	4405	3987	95.4	4179	100	4176	4388	3984	95.4	4168	98	4166
R_Date KHA_2010.005	4405	3983	95.3	4169	102	4166	4388	3979	95.4	4158	100	4156
R_Date K14_2000.077	4401	3980	95.4	4156	97	4153	4388	3976	95.5	4147	94	4144
R_Date KUR_2002.001	4405	3976	95.3	4153	101	4149	4400	3971	95.3	4142	99	4139
R_Date OBK_1971.004.02	4350	3980	95.4	4149	88	4146	4295	3979	95.4	4141	86	4139
R_Date KUR_2003.019	4380	3929	95.3	4129	97	4126	4347	3927	95.4	4120	94	4118
R_Date KUR_2002.006	4385	3926	95.4	4127	101	4123	4349	3923	95.4	4117	98	4115
R_Date SHM_1973.001	4346	3913	95.4	4111	99	4109	4290	3912	95.4	4102	96	4101
R_Date KHA_2010.009	4294	3910	95.4	4107	97	4106	4288	3912	95.4	4099	94	4098
R_Date OBK_1971.014.02	4245	3929	95.4	4098	82	4099	4243	3929	95.4	4092	80	4093
R_Date KUR_2002.015	4289	3910	95.4	4101	96	4099	4285	3906	95.4	4093	93	4092
R_Date SMS_1986.013	4287	3904	95.4	4095	95	4093	4282	3901	95.4	4088	92	4086
R_Date OBK_1971.014.01	4240	3929	95.4	4089	80	4090	4239	3929	95.4	4084	79	4085
R_Date KHA_2010.008	4238	3895	95.4	4062	90	4058	4233	3895	95.4	4056	88	4051
R_Date MNZ_1974.004.01	4225	3899	95.4	4049	75	4045	4224	3896	95.4	4045	73	4041
R_Date UIA_1977.005	4217	3888	95.4	4030	73	4030	4155	3885	95.4	4027	72	4027
R_Date K14_1998.037.01	4228	3865	95.4	4031	86	4030	4225	3856	95.4	4026	84	4025
R_Date KUR_2002.016	4218	3850	95.4	4017	83	4016	4155	3849	95.4	4013	81	4012
R_Date KUR_2002.004	4150	3849	95.4	4001	80	3999	4148	3852	95.4	3998	78	3995
R_Date KHA_2010.012	4150	3842	95.4	3998	84	3995	4149	3843	95.4	3995	82	3991
R_Date SHM_1973.002	4150	3837	95.4	3989	85	3984	4148	3839	95.4	3987	82	3981
R_Date UIA_surface	4138	3837	95.4	3967	74	3961	4091	3837	95.4	3966	72	3959
R_Date K14_1999.057.02	4148	3829	95.4	3966	85	3960	4146	3830	95.4	3965	81	3959
R_Date K14_2001.087	4148	3778	95.4	3963	86	3958	4147	3827	95.4	3963	82	3956
R_Date SHM_1972.002	4084	3842	95.4	3957	66	3949	4084	3843	95.4	3956	64	3948
R_Date UKH_1930.000	4089	3735	95.4	3938	74	3933	4086	3830	95.4	3938	71	3933

R_Date KHA_2003.003	4090	3726	95.5	3931	83	3928	4090	3738	95.3	3933	78	3928
R_Date OBK_1971.005	4085	3730	95.4	3922	76	3919	4085	3773	95.4	3925	72	3920
R_Date MNZ_1974.001	4080	3724	95.4	3903	76	3902	4080	3732	95.4	3908	70	3904
R_Date SMS_1987.021	4084	3720	95.4	3902	87	3901	4082	3731	95.4	3909	79	3905
R_Date SHA_2000.009	4086	3712	95.4	3900	99	3900	4083	3732	95.4	3911	88	3907
R_Date SHM_1972.001.01	4080	3702	95.4	3883	90	3885	4079	3726	95.4	3895	81	3892
R_Date K14_1998.036.01	4080	3702	95.4	3883	90	3885	4079	3726	95.4	3894	80	3891
R_Date SHA_2008.103.02	4084	3694	95.4	3878	102	3879	4082	3724	95.4	3895	90	3890
R_Date SHM_1973.004	4079	3697	95.4	3873	90	3875	4075	3724	95.4	3886	80	3883
R_Date KHA_2010.007	4065	3649	95.4	3844	88	3848	4064	3715	95.4	3863	77	3862
R_Date K14_1999.045	3980	3645	95.4	3821	84	3824	3980	3711	95.4	3845	73	3844
R_Date BO1_1971.002	3971	3693	95.4	3818	74	3823	3970	3718	95.4	3837	65	3839
R_Date K14_1998.037.02	3975	3649	95.4	3818	80	3821	3975	3716	95.4	3841	70	3841
R_Date OBK_1971.010	3967	3651	95.4	3812	72	3814	3967	3716	95.4	3832	63	3834
R_Date SHA_2008.107	3984	3637	95.4	3812	92	3810	3985	3699	95.4	3844	77	3840
R_Date BO2_1971.002	3878	3650	95.4	3776	52	3775	3880	3710	95.4	3795	45	3795
R_Date K14_1999.049	3964	3634	95.4	3780	79	3779	3965	3697	95.4	3816	64	3814
R_Date BO1_1971.001	3900	3641	95.4	3775	67	3774	3910	3697	95.4	3805	55	3804
R_Date GLZ_1887.006	3971	3608	95.4	3778	91	3776	3971	3698	95.4	3823	71	3818
R_Date KHA_2010.006	3920	3612	95.4	3767	78	3767	3957	3695	95.4	3808	61	3806
R_Date SHA_2008.109	3965	3580	95.4	3765	93	3764	3971	3694	95.4	3818	70	3813
R_Date SHA_2008.103.01	3958	3584	95.4	3760	90	3760	3968	3691	95.4	3813	67	3809
R_Date BO2_1971.003	3872	3614	95.4	3748	67	3750	3886	3692	95.4	3790	49	3794
R_Date SHM_1973.003.01	3890	3590	95.4	3747	78	3748	3911	3685	95.4	3798	56	3799
R_Date UID_1994.048	3887	3496	95.4	3705	93	3701	3909	3665	95.4	3792	58	3796
R_Date SHA_2008.111	3841	3478	95.4	3670	96	3666	3894	3661	95.4	3784	56	3793
Span EBA dated events							1101	1312	95.4	1200	53	1195
Interval EBA							613	924	95.4	760	82	753
Boundary EBA End							3800	3670	95.4	3735	32	3736
Start							3919	3687	95.4	3790	59	3782
Transition							0	298	95.4	110	91	88
End							3775	3570	95.4	3680	51	3687
Sequence Cis-Baikal IA n=47												
Boundary IA Start							2826	2520	95.4	2675	82	2675
Start							3026	2601	95.4	2834	99	2834
Transition							0	726	95.4	318	223	299
End							2808	2193	95.4	2516	168	2529
Phase												

R_Date Ua-49659	2307	1819	95.4	2024	120	2016	2307	1830	95.4	2043	115	2034
R_Date Ua-49660	2333	1901	95.4	2120	118	2114	2335	1927	95.4	2130	115	2124
R_Date Ua-49630	2315	1882	95.4	2086	119	2078	2315	1895	95.4	2098	116	2089
R_Date Ua-49628	1970	1558	95.4	1766	107	1768	2040	1690	95.4	1856	83	1855
R_Date Ua-49622	2359	1948	95.4	2192	113	2198	2359	1951	95.4	2196	108	2202
R_Date Ua-49623	2765	2355	95.4	2582	118	2583	2737	2356	95.4	2542	107	2539
R_Date Ua-49624	2698	2042	95.4	2303	143	2291	2681	2041	95.4	2294	131	2284
R_Date Ua-49625	2485	1994	95.4	2239	122	2233	2459	1999	95.4	2238	114	2234
R_Date Ua-49626	1970	1556	95.4	1762	106	1765	2041	1688	95.4	1854	83	1853
R_Date Ua-49627	2705	2132	95.4	2375	156	2357	2691	2126	95.4	2357	143	2346
R_Date Ua-49670	2705	2119	95.4	2356	154	2339	2689	2118	95.4	2340	141	2332
R_Date Ua-49648	2361	2013	95.4	2234	85	2233	2360	2045	95.4	2235	83	2234
R_Date Ua-49649	2750	2380	95.4	2587	99	2587	2738	2378	95.4	2558	95	2554
R_Date Ua-49650	2684	2149	95.4	2341	120	2337	2676	2149	95.4	2332	110	2335
R_Date Ua-49653	2308	1950	95.4	2118	97	2106	2310	1952	95.4	2124	96	2111
R_Date Ua-49655	2462	2129	95.4	2279	93	2274	2458	2137	95.4	2277	88	2273
R_Date Ua-49656	2706	2341	95.4	2496	113	2468	2696	2336	95.4	2474	103	2450
R_Date Ua-49657	2348	2061	95.4	2218	75	2223	2348	2062	95.4	2220	74	2224
R_Date Ua-49633	2705	2333	95.4	2486	114	2457	2696	2329	95.4	2465	104	2441
R_Date Ua-49634	2700	2161	95.4	2419	120	2401	2691	2160	95.4	2404	109	2392
R_Date Ua-49635	2746	2379	95.4	2579	100	2580	2723	2365	95.4	2550	96	2548
R_Date Ua-49631	2706	2345	95.4	2504	112	2478	2695	2342	95.4	2481	103	2457
R_Date Ua-49643	2711	2352	95.4	2526	111	2514	2697	2349	95.4	2501	104	2482
R_Date Ua-49638	2362	2116	95.4	2250	73	2242	2360	2119	95.4	2250	72	2243
R_Date Ua-49639	2763	2380	95.4	2606	98	2605	2751	2378	95.3	2573	93	2565
R_Date Ua-49641	2695	2153	95.4	2374	119	2357	2682	2153	95.4	2363	109	2353
R_Date Ua-49642	2736	2378	95.4	2564	103	2568	2711	2361	95.4	2538	98	2535
R_Date Ua-49637	2043	1733	95.4	1904	73	1904	2106	1802	95.4	1922	67	1919
R_Date Ua-49636	1985	1715	95.4	1844	69	1848	1991	1749	95.4	1873	59	1872
R_Date Ua-49662	2711	2353	95.4	2528	110	2517	2696	2350	95.4	2503	103	2484
R_Date Ua-49663	2460	2141	95.4	2279	90	2276	2456	2142	95.4	2277	86	2276
R_Date Ua-49652	2309	1949	95.4	2118	98	2107	2310	1952	95.4	2124	98	2112
R_Date Ua-49632	2759	2380	95.4	2603	98	2600	2749	2380	95.4	2571	93	2563
R_Date Ua-49645	2724	2359	95.4	2551	108	2554	2706	2355	95.4	2524	102	2517
R_Date Ua-49646	2376	2068	95.4	2249	76	2242	2367	2070	95.4	2249	74	2242
R_Date Ua-49647	2329	2004	95.4	2179	91	2180	2330	2005	95.4	2183	90	2184
R_Date Ua-49658	2945	2733	95.4	2812	56	2807	2861	2496	95.4	2730	97	2762
R_Date Ua-49664	1990	1709	95.4	1841	75	1845	1994	1739	95.4	1875	64	1873

R_Date Ua-49665	2320	2002	95.4	2171	92	2168	2321	2005	95.4	2175	91	2173
R_Date Ua-49666	2037	1715	95.4	1881	77	1883	2049	1749	95.4	1906	69	1903
R_Date Ua-49667	1987	1699	95.4	1826	73	1831	1988	1738	95.4	1862	62	1863
R_Date Ua-49668	2704	2180	95.4	2436	124	2415	2695	2164	95.3	2418	113	2404
R_Date Ua-49669	2304	1899	95.4	2074	92	2065	2305	1901	95.3	2080	93	2071
R_Date Ua-49671	2329	2005	95.4	2183	89	2184	2331	2006	95.4	2186	88	2188
R_Date Ua-49672	2353	2068	95.4	2231	72	2231	2353	2070	95.4	2232	71	2232
R_Date Ua-49654	2055	1606	95.4	1830	111	1832	2110	1721	95.4	1896	92	1889
R_Date OxA-21498	2849	2438	95.4	2647	110	2662	2777	2380	95.3	2593	101	2577
Span IA dated events							768	1135	95.4	968	87	973
Interval IA							563	1071	95.4	823	135	828
Boundary IA End							2029	1700	95.4	1851	83	1841
Start							2395	1722	95.4	2012	194	1965
Transition							0	795	95.4	322	257	271
End							1855	1496	95.4	1691	95	1702
Difference Gap							891	1228	95.4	1060	87	1060

Table S7. Oxcal (57, 58) output for Early Bronze Age (15) and Early Iron Age human radiocarbon dates using the uniform distribution model and adjusted for the FRE using the general regression model for Cis-Baikal (19).

	Unmodelled age cal. BP						Modelled age cal. BP					
	**from**	**to**	**%**	**μ date**	**σ**	**Median date**	**from**	**to**	**%**	**μ HPD date**	**μ HPD σ**	**Median date**
Sequence Cis-Baikal EBA n=91												
Boundary EBA Start							4950	4855	95.4	4897	24	4893
Phase												
R_Date SHM_1975.001	5276	4861	95.4	5014	110	4991	4922	4846	95.4	4880	18	4878
R_Date SMS_1986.009	5271	4827	95.5	4956	100	4937	4937	4742	95.4	4863	37	4866
R_Date UID_1991.042	5029	4570	95.4	4777	112	4784	4890	4575	95.4	4751	90	4749
R_Date GO2_1996.003	4959	4528	95.4	4737	102	4735	4873	4537	95.4	4726	91	4729
R_Date GO2_1996.004	4855	4455	95.4	4693	96	4697	4851	4525	95.4	4692	94	4697
R_Date GO2_1995.002	4845	4447	95.4	4674	100	4677	4844	4447	95.4	4673	100	4677
R_Date OBK_1976.003	4826	4530	95.4	4678	89	4678	4826	4530	95.4	4678	89	4678
R_Date KUR_2002.009	4815	4425	95.4	4606	110	4588	4815	4426	95.4	4606	110	4588
R_Date OBK_1971.013	4806	4358	95.4	4513	91	4497	4805	4359	95.4	4513	91	4497
R_Date MAK_1992.018	4781	4259	95.4	4458	82	4464	4781	4260	95.4	4458	82	4464
R_Date KUR_2003.025	4529	4159	95.4	4374	94	4374	4529	4159	95.4	4374	94	4374
R_Date KUR_2003.026	4524	4160	95.4	4362	89	4363	4524	4160	95.4	4362	89	4363
R_Date MAK_1992.019	4516	4161	95.4	4346	76	4348	4516	4161	95.4	4346	76	4348
R_Date OBK_1971.003.02	4514	4159	95.4	4339	76	4341	4514	4159	95.4	4339	76	4341
R_Date BAD_1920.000	4520	4099	95.4	4320	102	4323	4520	4099	95.4	4320	102	4323
R_Date UBE_1957.002	4517	4100	95.4	4315	97	4319	4517	4099	95.4	4315	97	4319
R_Date OBK_1971.001.02	4421	4185	95.4	4327	59	4331	4422	4185	95.4	4327	59	4331
R_Date MAK_1992.013.00	4436	4155	95.4	4318	76	4323	4435	4155	95.4	4318	76	4323
R_Date KUR_2003.018	4435	4094	95.4	4289	90	4293	4435	4093	95.4	4289	90	4294
R_Date KHA_2010.011	4434	4093	95.4	4288	90	4292	4434	4093	95.4	4288	90	4292
R_Date OBK_1971.001.03	4419	4101	95.4	4279	83	4280	4419	4102	95.4	4279	83	4280
R_Date MKV_1973.001	4418	4100	95.4	4274	84	4275	4418	4100	95.4	4274	84	4275
R_Date OBK_1971.007	4417	4100	95.4	4274	84	4274	4417	4100	95.4	4274	84	4274
R_Date KHA_2010.015	4421	4090	95.4	4266	94	4268	4421	4091	95.4	4266	94	4268
R_Date KUR_2002.007.01	4425	4084	95.4	4260	98	4261	4425	4084	95.4	4260	98	4261
R_Date KUL_1977.000	4420	4084	95.4	4248	97	4247	4420	4084	95.4	4248	97	4247
R_Date KUR_2002.013	4419	4014	95.4	4243	98	4240	4419	4016	95.4	4243	97	4240
R_Date KUR_2002.014	4417	4013	95.4	4236	97	4233	4417	4013	95.4	4236	97	4233
R_Date OBK_1971.001.01	4407	4091	95.4	4228	82	4221	4406	4091	95.4	4228	82	4221

R_Date KUR_2002.010	4415	4010	95.4	4228	97	4223	4415	4010	95.4	4227	97	4223
R_Date KUR_2002.003	4413	3999	95.4	4214	102	4210	4413	3999	95.4	4214	102	4210
R_Date KUR_2002.012	4411	3994	95.4	4203	103	4200	4410	3994	95.4	4203	103	4200
R_Date KUR_2002.005	4410	3992	95.4	4198	102	4196	4410	3992	95.4	4198	102	4196
R_Date OBK_1971.004.01	4406	3999	95.4	4195	90	4192	4406	4000	95.4	4195	90	4192
R_Date UID_1991.039	4413	3985	95.4	4193	115	4192	4412	3985	95.4	4193	115	4192
R_Date KUR_2002.007.02	4409	3990	95.4	4192	104	4190	4408	3990	95.4	4192	104	4190
R_Date SMS_1986.012	4408	3991	95.4	4190	102	4188	4408	3991	95.4	4190	102	4188
R_Date OBK_1971.003.01	4406	3994	95.4	4189	91	4186	4405	3994	95.4	4189	91	4186
R_Date KUR_2003.017	4406	3987	95.4	4180	100	4178	4405	3988	95.4	4180	100	4177
R_Date SMS_1987.033	4405	3987	95.4	4179	100	4176	4405	3987	95.4	4178	100	4176
R_Date KHA_2010.005	4405	3983	95.3	4169	102	4166	4403	3983	95.4	4169	102	4166
R_Date K14_2000.077	4401	3980	95.4	4156	97	4153	4400	3979	95.4	4156	96	4152
R_Date KUR_2002.001	4405	3976	95.3	4153	101	4149	4404	3976	95.4	4152	101	4148
R_Date OBK_1971.004.02	4350	3980	95.4	4149	88	4146	4350	3980	95.4	4148	87	4146
R_Date KUR_2003.019	4380	3929	95.3	4129	97	4126	4381	3929	95.4	4129	97	4126
R_Date KUR_2002.006	4385	3926	95.4	4127	101	4123	4385	3926	95.4	4127	100	4123
R_Date SHM_1973.001	4346	3913	95.4	4111	99	4109	4345	3911	95.4	4111	99	4109
R_Date KHA_2010.009	4294	3910	95.4	4107	97	4106	4293	3910	95.4	4107	97	4105
R_Date OBK_1971.014.02	4245	3929	95.4	4098	82	4099	4245	3929	95.4	4098	81	4099
R_Date KUR_2002.015	4289	3910	95.4	4101	96	4099	4289	3911	95.4	4101	96	4099
R_Date SMS_1986.013	4287	3904	95.4	4095	95	4093	4286	3905	95.4	4095	95	4093
R_Date OBK_1971.014.01	4240	3929	95.4	4089	80	4090	4240	3930	95.4	4089	80	4090
R_Date KHA_2010.008	4238	3895	95.4	4062	90	4058	4237	3896	95.4	4062	90	4058
R_Date MNZ_1974.004.01	4225	3899	95.4	4049	75	4045	4226	3899	95.4	4049	74	4045
R_Date UIA_1977.005	4217	3888	95.4	4030	73	4030	4216	3887	95.4	4030	73	4030
R_Date K14_1998.037.01	4228	3865	95.4	4031	86	4030	4228	3865	95.3	4031	86	4030
R_Date KUR_2002.016	4218	3850	95.4	4017	83	4016	4217	3850	95.4	4016	83	4016
R_Date KUR_2002.004	4150	3849	95.4	4001	80	3999	4149	3852	95.4	4001	80	3999
R_Date KHA_2010.012	4150	3842	95.4	3998	84	3995	4150	3843	95.4	3998	84	3994
R_Date SHM_1973.002	4150	3837	95.4	3989	85	3984	4150	3837	95.4	3989	85	3984
R_Date UIA_surface	4138	3837	95.4	3967	74	3961	4137	3837	95.4	3967	73	3961
R_Date K14_1999.057.02	4148	3829	95.4	3966	85	3960	4148	3828	95.4	3966	84	3960
R_Date K14_2001.087	4148	3778	95.4	3963	86	3958	4148	3777	95.4	3964	85	3958
R_Date SHM_1972.002	4084	3842	95.4	3957	66	3949	4084	3843	95.4	3957	65	3949
R_Date UKH_1930.000	4089	3735	95.4	3938	74	3933	4089	3777	95.4	3938	73	3933
R_Date KHA_2003.003	4090	3726	95.5	3931	83	3928	4090	3729	95.4	3932	81	3928
R_Date OBK_1971.005	4085	3730	95.4	3922	76	3919	4085	3733	95.3	3923	75	3920

R_Date MNZ_1974.001	4080	3724	95.4	3903	76	3902	4080	3726	95.3	3905	74	3903
R_Date SMS_1987.021	4084	3720	95.4	3902	87	3901	4083	3724	95.4	3904	84	3902
R_Date SHA_2000.009	4086	3712	95.4	3900	99	3900	4084	3723	95.4	3905	94	3903
R_Date SHM_1972.001.01	4080	3702	95.4	3883	90	3885	4080	3718	95.4	3888	86	3887
R_Date K14_1998.036.01	4080	3702	95.4	3883	90	3885	4080	3717	95.4	3888	86	3887
R_Date SHA_2008.103.02	4084	3694	95.4	3878	102	3879	4083	3710	95.4	3887	96	3884
R_Date SHM_1973.004	4079	3697	95.4	3873	90	3875	4076	3713	95.4	3878	85	3878
R_Date KHA_2010.007	4065	3649	95.4	3844	88	3848	4062	3701	95.4	3853	82	3854
R_Date K14_1999.045	3980	3645	95.4	3821	84	3824	3975	3701	95.4	3833	77	3834
R_Date BO1_1971.002	3971	3693	95.4	3818	74	3823	3966	3705	95.4	3826	68	3829
R_Date K14_1998.037.02	3975	3649	95.4	3818	80	3821	3971	3702	95.4	3829	73	3830
R_Date OBK_1971.010	3967	3651	95.4	3812	72	3814	3965	3701	95.4	3820	66	3823
R_Date SHA_2008.107	3984	3637	95.4	3812	92	3810	3982	3693	95.4	3831	81	3827
R_Date BO2_1971.002	3878	3650	95.4	3776	52	3775	3872	3700	95.4	3785	46	3783
R_Date K14_1999.049	3964	3634	95.4	3780	79	3779	3961	3691	95.4	3803	66	3797
R_Date BO1_1971.001	3900	3641	95.4	3775	67	3774	3902	3691	95.4	3793	56	3789
R_Date GLZ_1887.006	3971	3608	95.4	3778	91	3776	3971	3688	95.4	3810	74	3802
R_Date KHA_2010.006	3920	3612	95.4	3767	78	3767	3957	3685	95.4	3795	62	3791
R_Date SHA_2008.109	3965	3580	95.4	3765	93	3764	3969	3683	95.4	3804	72	3797
R_Date SHA_2008.103.01	3958	3584	95.4	3760	90	3760	3965	3681	95.4	3800	69	3794
R_Date BO2_1971.003	3872	3614	95.4	3748	67	3750	3880	3685	95.4	3778	50	3779
R_Date SHM_1973.003.01	3890	3590	95.4	3747	78	3748	3901	3680	95.4	3785	57	3783
R_Date UID_1994.048	3887	3496	95.4	3705	93	3701	3896	3665	95.4	3779	59	3779
R_Date SHA_2008.111	3841	3478	95.4	3670	96	3666	3880	3661	95.4	3772	56	3773
Span EBA dated events							1104	1249	95.4	1173	35	1171
Boundary EBA End							3762	3630	95.4	3698	32	3701
Sequence Cis-Baikal IA n=47												
Boundary IA Start							2774	2513	95.4	2654	68	2661
Phase												
R_Date Ua-49659	2307	1819	95.4	2024	120	2016	2305	1826	95.4	2033	113	2022
R_Date Ua-49660	2333	1901	95.4	2120	118	2114	2332	1904	95.4	2121	116	2115
R_Date Ua-49630	2315	1882	95.4	2086	119	2078	2311	1890	95.4	2088	117	2079
R_Date Ua-49628	1970	1558	95.4	1766	107	1768	2038	1734	95.4	1877	71	1871
R_Date Ua-49622	2359	1948	95.4	2192	113	2198	2357	1950	95.4	2191	110	2197
R_Date Ua-49623	2765	2355	95.4	2582	118	2583	2693	2352	95.4	2519	93	2519
R_Date Ua-49624	2698	2042	95.4	2303	143	2291	2671	2041	95.4	2291	129	2283
R_Date Ua-49625	2485	1994	95.4	2239	122	2233	2460	1998	95.4	2234	114	2232
R_Date Ua-49626	1970	1556	95.4	1762	106	1765	2037	1734	95.4	1876	70	1870

R_Date Ua-49627	2705	2132	95.4	2375	156	2357	2677	2126	95.4	2353	139	2346
R_Date Ua-49670	2705	2119	95.4	2356	154	2339	2678	2117	95.4	2336	137	2332
R_Date Ua-49648	2696	1998	95.3	2282	148	2264	2655	1997	95.4	2270	134	2259
R_Date Ua-49649	2724	2159	95.4	2460	146	2448	2686	2158	95.4	2427	129	2422
R_Date Ua-49650	2709	2150	95.4	2402	157	2389	2682	2145	95.4	2375	139	2370
R_Date Ua-49653	2315	1880	95.4	2084	119	2075	2311	1890	95.4	2086	117	2077
R_Date Ua-49655	2705	2070	95.3	2343	153	2328	2679	2068	95.3	2325	137	2321
R_Date Ua-49656	2707	2149	95.4	2396	156	2382	2681	2143	95.4	2370	139	2364
R_Date Ua-49657	2705	2116	95.4	2348	154	2333	2679	2113	95.4	2329	137	2326
R_Date Ua-49633	2778	2357	95.4	2591	119	2591	2692	2354	95.4	2523	91	2524
R_Date Ua-49634	2744	2326	95.4	2519	126	2513	2708	2310	95.4	2478	109	2470
R_Date Ua-49635	2744	2329	95.4	2520	125	2515	2706	2312	95.4	2479	109	2471
R_Date Ua-49631	2744	2212	95.4	2506	130	2496	2705	2188	95.4	2467	114	2459
R_Date Ua-49643	2705	2069	95.4	2342	154	2327	2678	2067	95.3	2323	137	2320
R_Date Ua-49638	2705	2133	95.4	2375	156	2356	2677	2127	95.4	2352	138	2346
R_Date Ua-49639	2775	2356	95.4	2588	119	2588	2692	2353	95.4	2522	92	2522
R_Date Ua-49641	2738	2184	95.4	2487	137	2474	2697	2180	95.4	2450	121	2444
R_Date Ua-49642	2745	2355	95.4	2556	117	2559	2694	2346	95.4	2505	98	2503
R_Date Ua-49637	2060	1611	95.4	1839	112	1841	2110	1757	95.4	1909	83	1899
R_Date Ua-49636	2119	1633	95.4	1885	111	1885	2120	1776	95.4	1931	88	1921
R_Date Ua-49662	2743	2344	95.4	2534	122	2534	2702	2331	95.4	2490	104	2484
R_Date Ua-49663	2705	2119	95.4	2356	155	2340	2678	2117	95.4	2336	138	2332
R_Date Ua-49652	2701	2062	95.4	2324	150	2312	2676	2060	95.4	2309	134	2306
R_Date Ua-49632	2845	2365	95.4	2627	126	2627	2700	2358	95.4	2533	88	2534
R_Date Ua-49645	2750	2340	95.4	2538	124	2540	2705	2330	95.4	2491	105	2487
R_Date Ua-49646	2705	2115	95.4	2347	154	2332	2678	2071	95.4	2328	137	2324
R_Date Ua-49647	2699	2044	95.4	2308	146	2296	2671	2042	95.5	2294	131	2287
R_Date Ua-49658	2745	2351	95.4	2548	119	2551	2696	2343	95.4	2500	100	2497
R_Date Ua-49664	2002	1569	95.4	1807	109	1809	2060	1741	95.4	1894	77	1885
R_Date Ua-49665	2333	1903	95.4	2122	117	2116	2332	1924	95.4	2123	116	2116
R_Date Ua-49666	2101	1611	95.4	1841	115	1842	2111	1759	95.4	1912	85	1902
R_Date Ua-49667	2110	1623	95.4	1861	114	1861	2114	1770	95.4	1920	86	1910
R_Date Ua-49668	2712	2153	95.4	2416	156	2404	2680	2150	95.4	2387	138	2384
R_Date Ua-49669	2315	1876	95.4	2079	120	2071	2310	1884	95.4	2082	117	2072
R_Date Ua-49671	2300	1734	95.4	1986	118	1978	2301	1816	95.4	2001	107	1988
R_Date Ua-49672	2307	1822	95.4	2034	121	2026	2306	1833	95.3	2041	114	2031
R_Date Ua-49654	2055	1606	95.4	1830	111	1832	2107	1752	95.4	1905	81	1895
R_Date OxA-21498	2745	2356	95.4	2557	116	2561	2691	2348	95.4	2507	97	2505

Span IA dated events	637	983	95.4	815	88	820
Boundary EIA End	1904	1691	95.4	1803	53	1808

Table S8. Oxcal (Bronk Ramsey 57, 58) output for Early Bronze Age (15) and Early Iron Age human radiocarbon dates using the uniform distribution model and adjusted for the FRE using the general regression model for Cis-Baikal (19). In the first run of the model, date Ua-49658 was identified as being in poor agreement with the other dates. This date was removed and the model ran a second time, producing the results shown here.

	Unmodelled age cal. BP						Modelled age cal. BP					
	from	to	%	μ date	σ	Median date	from	to	%	μ HPD date	μ HPD σ	Median date
Sequence Cis-Baikal EBA n=91												
Boundary EBA Start							4950	4855	95.4	4897	24	4893
Phase												
R_Date SHM_1975.001	5276	4861	95.4	5014	110	4991	4922	4846	95.4	4880	18	4878
R_Date SMS_1986.009	5271	4827	95.5	4956	100	4937	4937	4744	95.4	4863	37	4866
R_Date UID_1991.042	5029	4570	95.4	4777	112	4784	4890	4575	95.4	4750	90	4748
R_Date GO2_1996.003	4959	4528	95.4	4737	102	4735	4873	4538	95.4	4726	91	4729
R_Date GO2_1996.004	4855	4455	95.4	4693	96	4697	4851	4525	95.4	4691	94	4696
R_Date GO2_1995.002	4845	4447	95.4	4674	100	4677	4844	4447	95.4	4673	100	4677
R_Date OBK_1976.003	4826	4530	95.4	4678	89	4678	4826	4530	95.4	4678	89	4679
R_Date KUR_2002.009	4815	4425	95.4	4606	110	4588	4815	4426	95.4	4606	110	4588
R_Date OBK_1971.013	4806	4358	95.4	4513	91	4497	4805	4358	95.3	4513	91	4496
R_Date MAK_1992.018	4781	4259	95.4	4458	82	4464	4781	4260	95.4	4458	82	4464
R_Date KUR_2003.025	4529	4159	95.4	4374	94	4374	4529	4159	95.4	4374	94	4374
R_Date KUR_2003.026	4524	4160	95.4	4362	89	4363	4524	4160	95.4	4362	89	4363
R_Date MAK_1992.019	4516	4161	95.4	4346	76	4348	4516	4161	95.4	4346	76	4348
R_Date OBK_1971.003.02	4514	4159	95.4	4339	76	4341	4514	4159	95.4	4339	76	4341
R_Date BAD_1920.000	4520	4099	95.4	4320	102	4323	4520	4098	95.3	4320	102	4323
R_Date UBE_1957.002	4517	4100	95.4	4315	97	4319	4517	4100	95.4	4315	97	4319
R_Date OBK_1971.001.02	4421	4185	95.4	4327	59	4331	4422	4185	95.4	4327	59	4331
R_Date MAK_1992.013.00	4436	4155	95.4	4318	76	4323	4435	4155	95.4	4318	76	4323
R_Date KUR_2003.018	4435	4094	95.4	4289	90	4293	4435	4094	95.4	4289	90	4294
R_Date KHA_2010.011	4434	4093	95.4	4288	90	4292	4435	4093	95.4	4288	90	4292
R_Date OBK_1971.001.03	4419	4101	95.4	4279	83	4280	4419	4102	95.4	4279	83	4280
R_Date MKV_1973.001	4418	4100	95.4	4274	84	4275	4418	4099	95.4	4274	84	4275
R_Date OBK_1971.007	4417	4100	95.4	4274	84	4274	4418	4100	95.4	4274	83	4274
R_Date KHA_2010.015	4421	4090	95.4	4266	94	4268	4421	4091	95.4	4266	94	4267
R_Date KUR_2002.007.01	4425	4084	95.4	4260	98	4261	4425	4083	95.4	4260	98	4261
R_Date KUL_1977.000	4420	4084	95.4	4248	97	4247	4420	4084	95.4	4248	97	4247
R_Date KUR_2002.013	4419	4014	95.4	4243	98	4240	4419	4014	95.4	4243	98	4240
R_Date KUR_2002.014	4417	4013	95.4	4236	97	4233	4417	4013	95.4	4236	97	4233

R_Date OBK_1971.001.01	4407	4091	95.4	4228	82	4221	4406	4091	95.4	4229	82	4221
R_Date KUR_2002.010	4415	4010	95.4	4228	97	4223	4415	4010	95.4	4228	97	4223
R_Date KUR_2002.003	4413	3999	95.4	4214	102	4210	4413	3999	95.4	4214	102	4210
R_Date KUR_2002.012	4411	3994	95.4	4203	103	4200	4410	3994	95.4	4203	103	4200
R_Date KUR_2002.005	4410	3992	95.4	4198	102	4196	4410	3993	95.4	4198	102	4196
R_Date OBK_1971.004.01	4406	3999	95.4	4195	90	4192	4405	4000	95.5	4195	90	4192
R_Date UID_1991.039	4413	3985	95.4	4193	115	4192	4412	3985	95.4	4194	115	4192
R_Date KUR_2002.007.02	4409	3990	95.4	4192	104	4190	4409	3990	95.4	4192	104	4190
R_Date SMS_1986.012	4408	3991	95.4	4190	102	4188	4407	3991	95.4	4190	102	4188
R_Date OBK_1971.003.01	4406	3994	95.4	4189	91	4186	4405	3994	95.4	4189	91	4186
R_Date KUR_2003.017	4406	3987	95.4	4180	100	4178	4405	3988	95.4	4180	100	4178
R_Date SMS_1987.033	4405	3987	95.4	4179	100	4176	4405	3987	95.3	4179	100	4176
R_Date KHA_2010.005	4405	3983	95.3	4169	102	4166	4404	3983	95.4	4169	102	4166
R_Date K14_2000.077	4401	3980	95.4	4156	97	4153	4401	3980	95.3	4156	96	4153
R_Date KUR_2002.001	4405	3976	95.3	4153	101	4149	4403	3976	95.4	4153	101	4149
R_Date OBK_1971.004.02	4350	3980	95.4	4149	88	4146	4350	3980	95.4	4149	87	4146
R_Date KUR_2003.019	4380	3929	95.3	4129	97	4126	4379	3929	95.3	4129	97	4126
R_Date KUR_2002.006	4385	3926	95.4	4127	101	4123	4385	3926	95.5	4127	100	4124
R_Date SHM_1973.001	4346	3913	95.4	4111	99	4109	4345	3912	95.4	4111	99	4109
R_Date KHA_2010.009	4294	3910	95.4	4107	97	4106	4294	3910	95.4	4107	97	4105
R_Date OBK_1971.014.02	4245	3929	95.4	4098	82	4099	4245	3930	95.4	4098	81	4099
R_Date KUR_2002.015	4289	3910	95.4	4101	96	4099	4289	3911	95.4	4101	96	4099
R_Date SMS_1986.013	4287	3904	95.4	4095	95	4093	4287	3905	95.4	4095	95	4093
R_Date OBK_1971.014.01	4240	3929	95.4	4089	80	4090	4240	3929	95.4	4089	80	4090
R_Date KHA_2010.008	4238	3895	95.4	4062	90	4058	4237	3895	95.4	4062	90	4057
R_Date MNZ_1974.004.01	4225	3899	95.4	4049	75	4045	4226	3900	95.4	4049	74	4045
R_Date UIA_1977.005	4217	3888	95.4	4030	73	4030	4216	3888	95.4	4030	73	4030
R_Date K14_1998.037.01	4228	3865	95.4	4031	86	4030	4228	3865	95.4	4031	86	4030
R_Date KUR_2002.016	4218	3850	95.4	4017	83	4016	4217	3852	95.4	4017	83	4016
R_Date KUR_2002.004	4150	3849	95.4	4001	80	3999	4150	3852	95.4	4001	80	3999
R_Date KHA_2010.012	4150	3842	95.4	3998	84	3995	4150	3843	95.4	3998	84	3995
R_Date SHM_1973.002	4150	3837	95.4	3989	85	3984	4150	3837	95.4	3989	85	3985
R_Date UIA_surface	4138	3837	95.4	3967	74	3961	4137	3837	95.4	3968	73	3961
R_Date K14_1999.057.02	4148	3829	95.4	3966	85	3960	4148	3828	95.4	3966	84	3960
R_Date K14_2001.087	4148	3778	95.4	3963	86	3958	4148	3778	95.4	3964	85	3958
R_Date SHM_1972.002	4084	3842	95.4	3957	66	3949	4084	3843	95.4	3957	65	3949
R_Date UKH_1930.000	4089	3735	95.4	3938	74	3933	4089	3777	95.4	3938	73	3933
R_Date KHA_2003.003	4090	3726	95.5	3931	83	3928	4090	3729	95.4	3932	81	3928

R_Date OBK_1971.005	4085	3730	95.4	3922	76	3919	4084	3733	95.4	3923	75	3920
R_Date MNZ_1974.001	4080	3724	95.4	3903	76	3902	4080	3726	95.4	3905	74	3903
R_Date SMS_1987.021	4084	3720	95.4	3902	87	3901	4083	3724	95.4	3905	84	3903
R_Date SHA_2000.009	4086	3712	95.4	3900	99	3900	4084	3723	95.4	3905	94	3903
R_Date SHM_1972.001.01	4080	3702	95.4	3883	90	3885	4080	3718	95.4	3888	86	3887
R_Date K14_1998.036.01	4080	3702	95.4	3883	90	3885	4080	3718	95.4	3888	86	3887
R_Date SHA_2008.103.02	4084	3694	95.4	3878	102	3879	4083	3710	95.4	3887	95	3884
R_Date SHM_1973.004	4079	3697	95.4	3873	90	3875	4076	3713	95.4	3878	85	3878
R_Date KHA_2010.007	4065	3649	95.4	3844	88	3848	4061	3701	95.4	3853	82	3854
R_Date K14_1999.045	3980	3645	95.4	3821	84	3824	3975	3701	95.4	3833	77	3833
R_Date BO1_1971.002	3971	3693	95.4	3818	74	3823	3966	3705	95.4	3826	68	3829
R_Date K14_1998.037.02	3975	3649	95.4	3818	80	3821	3971	3703	95.4	3829	73	3830
R_Date OBK_1971.010	3967	3651	95.4	3812	72	3814	3965	3701	95.4	3821	66	3823
R_Date SHA_2008.107	3984	3637	95.4	3812	92	3810	3981	3694	95.4	3831	81	3827
R_Date BO2_1971.002	3878	3650	95.4	3776	52	3775	3873	3701	95.4	3785	46	3783
R_Date K14_1999.049	3964	3634	95.4	3780	79	3779	3961	3691	95.4	3803	66	3798
R_Date BO1_1971.001	3900	3641	95.4	3775	67	3774	3902	3691	95.4	3793	56	3789
R_Date GLZ_1887.006	3971	3608	95.4	3778	91	3776	3970	3688	95.4	3810	74	3803
R_Date KHA_2010.006	3920	3612	95.4	3767	78	3767	3957	3686	95.4	3795	62	3791
R_Date SHA_2008.109	3965	3580	95.4	3765	93	3764	3967	3684	95.4	3804	72	3797
R_Date SHA_2008.103.01	3958	3584	95.4	3760	90	3760	3965	3682	95.4	3800	69	3794
R_Date BO2_1971.003	3872	3614	95.4	3748	67	3750	3880	3685	95.4	3778	50	3779
R_Date SHM_1973.003.01	3890	3590	95.4	3747	78	3748	3900	3680	95.4	3785	57	3784
R_Date UID_1994.048	3887	3496	95.4	3705	93	3701	3896	3666	95.4	3779	59	3779
R_Date SHA_2008.111	3841	3478	95.4	3670	96	3666	3879	3662	95.4	3772	56	3774
Span EBA dated events							1104	1248	95.4	1172	35	1170
Boundary EBA End							3761	3631	95.4	3699	31	3701
Sequence Cis-Baikal IA n=47												
Boundary IA Start							2765	2528	95.4	2646	64	2644
Phase												
R_Date Ua-49659	2307	1819	95.4	2024	120	2016	2305	1825	95.4	2032	113	2021
R_Date Ua-49660	2333	1901	95.4	2120	118	2114	2332	1904	95.4	2121	116	2115
R_Date Ua-49630	2315	1882	95.4	2086	119	2078	2311	1889	95.4	2088	117	2078
R_Date Ua-49628	1970	1558	95.4	1766	107	1768	2034	1740	95.4	1876	67	1868
R_Date Ua-49622	2359	1948	95.4	2192	113	2198	2358	1950	95.4	2191	110	2197
R_Date Ua-49623	2765	2355	95.4	2582	118	2583	2686	2351	95.4	2515	90	2518
R_Date Ua-49624	2698	2042	95.4	2303	143	2291	2653	2010	95.4	2289	127	2281
R_Date Ua-49625	2485	1994	95.4	2239	122	2233	2459	1998	95.4	2234	113	2232

R_Date Ua-49626	1970	1556	95.4	1762	106	1765	2035	1740	95.4	1875	67	1868
R_Date Ua-49627	2705	2132	95.4	2375	156	2357	2668	2124	95.4	2350	136	2345
R_Date Ua-49670	2705	2119	95.4	2356	154	2339	2672	2116	95.4	2334	135	2331
R_Date Ua-49648	2361	2013	95.4	2234	85	2233	2361	2042	95.4	2233	83	2233
R_Date Ua-49649	2750	2380	95.4	2587	99	2587	2687	2360	95.4	2533	80	2534
R_Date Ua-49650	2684	2149	95.4	2341	120	2337	2655	2146	95.4	2330	106	2334
R_Date Ua-49653	2308	1950	95.4	2118	97	2106	2309	1950	95.4	2118	97	2106
R_Date Ua-49655	2462	2129	95.4	2279	93	2274	2459	2138	95.4	2276	87	2271
R_Date Ua-49656	2706	2341	95.4	2496	113	2468	2679	2334	95.4	2463	93	2445
R_Date Ua-49657	2348	2061	95.4	2218	75	2223	2348	2061	95.4	2218	75	2223
R_Date Ua-49633	2705	2333	95.4	2486	114	2457	2680	2327	95.4	2455	94	2438
R_Date Ua-49634	2700	2161	95.4	2419	120	2401	2679	2160	95.3	2399	101	2391
R_Date Ua-49635	2746	2379	95.4	2579	100	2580	2672	2358	95.4	2527	82	2529
R_Date Ua-49631	2706	2345	95.4	2504	112	2478	2680	2340	95.4	2470	92	2452
R_Date Ua-49643	2711	2352	95.4	2526	111	2514	2674	2347	95.4	2486	92	2473
R_Date Ua-49638	2362	2116	95.4	2250	73	2242	2360	2119	95.4	2249	72	2242
R_Date Ua-49639	2763	2380	95.4	2606	98	2605	2706	2364	95.4	2542	76	2542
R_Date Ua-49641	2695	2153	95.4	2374	119	2357	2673	2153	95.3	2360	104	2353
R_Date Ua-49642	2736	2378	95.4	2564	103	2568	2675	2356	95.4	2517	86	2518
R_Date Ua-49637	2043	1733	95.4	1904	73	1904	2059	1810	95.4	1919	62	1914
R_Date Ua-49636	1985	1715	95.4	1844	69	1848	1990	1785	95.4	1879	51	1876
R_Date Ua-49662	2711	2353	95.4	2528	110	2517	2671	2348	95.4	2488	91	2475
R_Date Ua-49663	2460	2141	95.4	2279	90	2276	2456	2143	95.4	2276	84	2274
R_Date Ua-49652	2309	1949	95.4	2118	98	2107	2309	1949	95.4	2118	98	2106
R_Date Ua-49632	2759	2380	95.4	2603	98	2600	2705	2363	95.4	2541	76	2542
R_Date Ua-49645	2724	2359	95.4	2551	108	2554	2674	2351	95.4	2505	90	2502
R_Date Ua-49646	2376	2068	95.4	2249	76	2242	2367	2069	95.4	2248	74	2241
R_Date Ua-49647	2329	2004	95.4	2179	91	2180	2328	2004	95.4	2179	91	2180
R_Date Ua-49664	1990	1709	95.4	1841	75	1845	1995	1774	95.4	1881	55	1877
R_Date Ua-49665	2320	2002	95.4	2171	92	2168	2320	2002	95.4	2171	92	2168
R_Date Ua-49666	2037	1715	95.4	1881	77	1883	2042	1786	95.4	1906	62	1901
R_Date Ua-49667	1987	1699	95.4	1826	73	1831	1990	1774	95.4	1873	52	1869
R_Date Ua-49668	2704	2180	95.4	2436	124	2415	2679	2179	95.4	2411	105	2403
R_Date Ua-49669	2304	1899	95.4	2074	92	2065	2304	1899	95.4	2074	92	2065
R_Date Ua-49671	2329	2005	95.4	2183	89	2184	2330	2005	95.4	2183	89	2184
R_Date Ua-49672	2353	2068	95.4	2231	72	2231	2353	2068	95.4	2231	71	2231
R_Date Ua-49654	2055	1606	95.4	1830	111	1832	2105	1759	95.4	1903	79	1892
R_Date OxA-21498	2849	2438	95.4	2647	110	2662	2727	2365	95.4	2548	75	2546

Span IA dated events	664	959	95.4	807	76	804
Boundary EIA End	1885	1705	95.4	1803	45	1809

Table S9. Oxcal (57, 58) output for radiocarbon dates on Early Iron Age domestic faunal remains from cemeteries and habitation sites.

a) Trapezium distribution model (60).

	**Unmodelled age cal. BP**						**Modelled age cal. BP**					
	**from**	**to**	**%**	**μ date**	**σ**	**Median date**	**from**	**to**	**%**	**μ HPD date**	**μ HPD σ**	**Median date**
Sequence EIA Fauna n=10												
Boundary EIA Fauna Start							3276	2605	95.4	2909	170	2882
Start							3589	2796	95.4	3092	244	3026
Transition							0	1068	95.4	365	341	273
End							3190	2194	95.4	2727	237	2759
Phase												
R_Date OxA23939	2923	2776	95.4	2837	35	2835	2918	2773	95.4	2829	33	2825
R_Date OxA23994	2680	2351	95.4	2443	83	2423	2680	2350	95.4	2442	82	2422
R_Date OxA23989	2037	1887	95.4	1956	35	1957	2039	1888	95.4	1959	35	1959
R_Date OxA22389	1883	1735	95.4	1824	41	1833	1890	1740	95.4	1835	36	1843
R_Date Ua-49674	2487	2327	95.4	2393	59	2376	2486	2328	95.4	2392	58	2376
R_Date Ua-49673	2735	2465	95.4	2591	79	2585	2735	2463	95.4	2588	78	2583
R_Date Ua-49677	2702	2355	95.3	2513	106	2483	2701	2355	95.5	2511	105	2481
R_Date Ua-49676	2770	2510	95.5	2710	64	2734	2769	2507	95.5	2707	67	2734
R_Date Ua-49675	2677	2341	95.4	2424	75	2408	2677	2341	95.4	2422	74	2407
R_Date Ua-53580	2687	2351	95.4	2460	93	2433	2687	2351	95.5	2458	91	2433
Span EBA dated events							908	1100	95.4	994	49	989
Interval EBA							596	1619	95.4	1076	282	1042
Boundary EIA Fauna End							2222	1440	95.4	1834	202	1838
Start							2779	1561	95.4	2111	334	2077
Transition							0	1339	95.4	555	427	502
End							1865	1045	95.4	1556	248	1621

b) Uniform distribution model (57, 58)

	**Unmodelled age cal. BP**						**Modelled age cal. BP**					
	**from**	**to**	**%**	**μ date**	**σ**	**Median date**	**from**	**to**	**%**	**μ HPD date**	**μ HPD σ**	**Median date**
Sequence EIA Fauna n=10												
Boundary Fauna EIA Start							3179	2779	95.4	2927	124	2894
Phase												
R_Date OxA23939	2923	2776	95.4	2837	35	2835	2884	2770	95.4	2825	31	2820
R_Date OxA23994	2680	2351	95.4	2443	83	2423	2680	2350	95.4	2443	83	2422
R_Date OxA23989	2037	1887	95.4	1956	35	1957	2037	1887	95.4	1956	35	1957

R_Date OxA22389	1883	1735	95.4	1824	41	1833	1894	1741	95.4	1839	34	1847
R_Date Ua-49674	2487	2327	95.4	2393	59	2376	2486	2327	95.4	2392	59	2375
R_Date Ua-49673	2735	2465	95.4	2591	79	2585	2735	2465	95.4	2591	78	2585
R_Date Ua-49677	2702	2355	95.3	2513	106	2483	2702	2355	95.5	2513	106	2483
R_Date Ua-49676	2770	2510	95.5	2710	64	2734	2770	2510	95.4	2711	63	2735
R_Date Ua-49675	2677	2341	95.4	2424	75	2408	2677	2341	95.4	2423	75	2407
R_Date Ua-53580	2687	2351	95.4	2460	93	2433	2688	2351	95.4	2459	93	2433
Span EIA dated events							903	1088	95.4	985	47	980
Interval Phase 1							934	1579	95.4	1191	189	1145
Boundary Fauna EIA End							1887	1476	95.4	1737	126	1771
